# Identification of differentially expressed lncRNAs involved in transient regeneration of the neonatal C57BL/6J mouse heart by next-generation high-throughput RNA sequencing

**DOI:** 10.18632/oncotarget.15887

**Published:** 2017-03-03

**Authors:** Yu-Mei Chen, Hua Li, Yi Fan, Qi-Jun Zhang, Xing Li, Li-Jie Wu, Zi-jie Chen, Chun Zhu, Ling-Mei Qian

**Affiliations:** ^1^ Department of Emergency, Zhongshan Hospital, Fudan University, Shanghai 200032, P.R. China; ^2^ Department of Cardiology, The First Affiliated Hospital of Nanjing Medical University, Nanjing 210029, P. R. China; ^3^ Department of Pediatrics, Obstetrics and Gynecology Hospital Affiliated to Nanjing Medical University, Nanjing, Jiangsu 210004, P. R. China

**Keywords:** neonatal mouse, heart, regeneration, lncRNAs

## Abstract

Previous studies have shown that mammalian cardiac tissue has a regenerative capacity. Remarkably, neonatal mice can regenerate their cardiac tissue for up to 6 days after birth, but this capacity is lost by day 7. In this study, we aimed to explore the expression pattern of long noncoding RNA (lncRNA) during this period and examine the mechanisms underlying this process. We found that 685 lncRNAs and 1833 mRNAs were differentially expressed at P1 and P7 by the next-generation high-throughput RNA sequencing. The coding genes associated with differentially expressed lncRNAs were mainly involved in metabolic processes and cell proliferation, and also were potentially associated with several key regeneration signalling pathways, including PI3K-Akt, MAPK, Hippo and Wnt. In addition, we identified some correlated targets of highly-dysregulated lncRNAs such as Igfbp3, Trnp1, Itgb6, and Pim3 by the coding-noncoding gene co-expression network. These data may offer a reference resource for further investigation about the mechanisms by which lncRNAs regulate cardiac regeneration.

## INTRODUCTION

In recent years, the traditional view that the heart is a terminally differentiated organ with no capacity for cardiomyocyte renewal has been emphatically refuted by an increasing number of studies in humans and other mammals [1–3]. Several studies have found that 1-day-old neonatal mice were capable of undergoing cardiac regeneration following multiple types of cardiac injury, but the ability to efficiently regenerate heart muscle was lost by postnatal day 7 [4–6]. Further study by our group examined the global gene expression patterns of the neonatal mouse heart at key time points (1 and 7 days old) [7] and found several differentially expressed genes involved in transient regeneration of the neonatal mouse heart. However, there is limited original data regarding cardiac regeneration during this developmental period.

Long noncoding RNAs (lncRNAs) are defined as transcripts greater than 200 nt without a known protein-coding function [8]. There is increasing evidence that lncRNAs can regulate gene expression at the epigenetic, transcription, and post-transcription levels and participate in a range of biological processes [9]. A recent study showed that many lncRNAs are expressed in the heart and confirmed the essential role of lncRNAs in the cardiovascular system such as modulating cardiac development [10]. Further studies have shown that lncRNAs are involved in coronary artery disease [11] and myocardial hypertrophy [12]. In addition, some lncRNAs have been found to be involved in cardiac regeneration. For example, lncRNA-ST8SIA3 (also named lncRNA ROR, regulator of reprogramming) enhances the reprogramming of fibroblasts to become induced pluripotent stem cells or cardiomyocytes [13]. Knocking out the gene lncRNA-H19 has been shown to promote differentiation of parthenogenetic embryonic stem cells to cardiomyocytes with strong heart-like beating [14]. However, our understanding of the role of lncRNAs in regeneration of heart tissue in mice is still limited. Therefore, two key time points (1 and 7 days old) were selected for the analysis of global lncRNA expression profiles in C57BL/6 mice using next-generation high-throughput RNA sequencing techniques.

## RESULTS

### Profiles of the differently expressed lncRNAs and mRNAs

**Figure 1:**
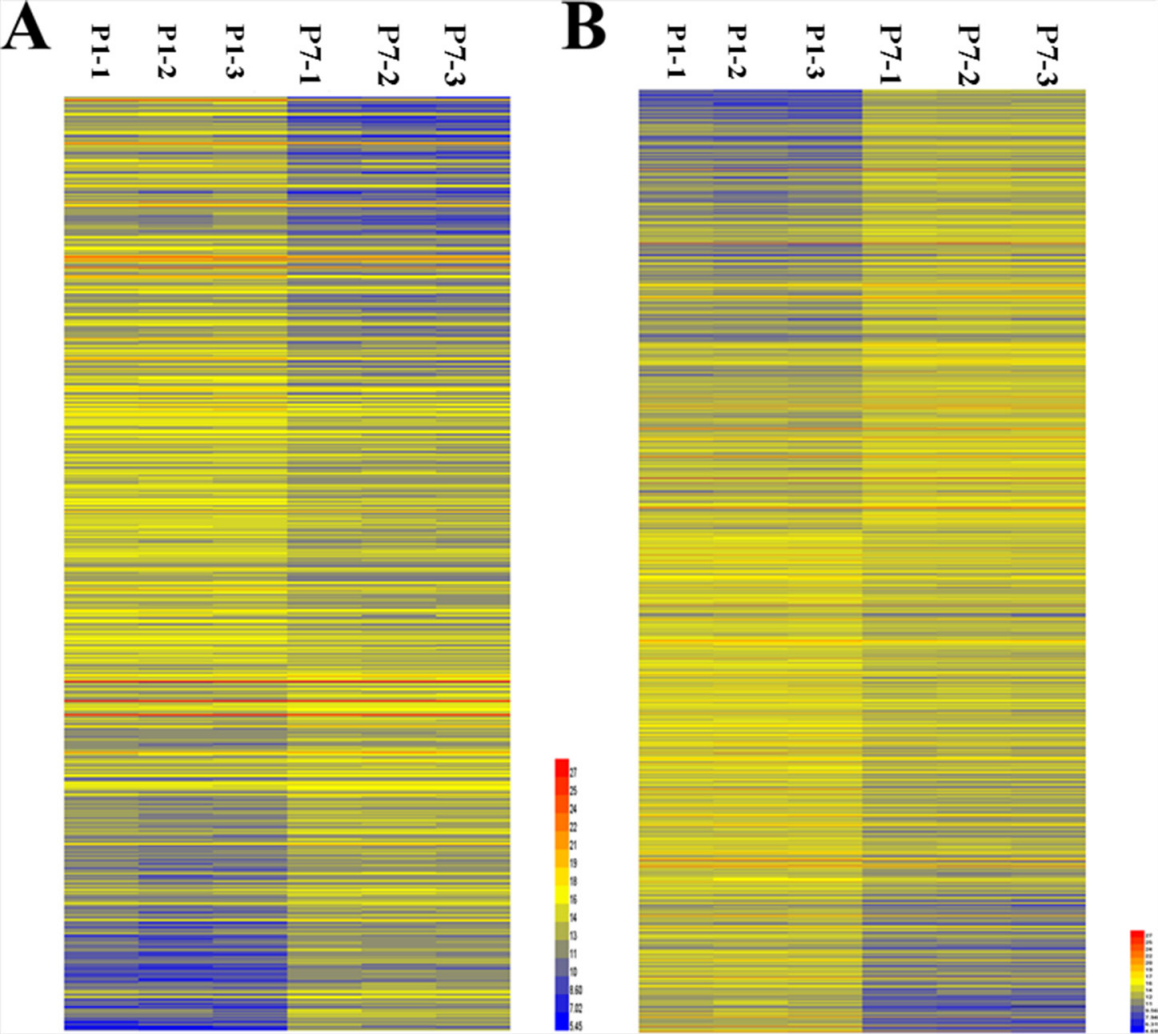
Hierarchical clustering of the differentially expressed LncRNAs and mRNAs in murine cardiac tissue in postnatal days 1 and 7 (P1 and P7) (**A**) Differentially expressed LncRNAs; (**B**) Differentially expressed mRNAs. Yellow indicates upregulation; blue indicates downregulation.

From the lncRNA expression profiles, 685 differentially expressed lncRNAs in mouse cardiac tissue were compared between P1 and P7. Compared to 1-day-old cardiac tissue, we identified 418 downregulated lncRNAs and 267 upregulated lncRNAs in 7-day-old cardiac tissue. As shown in Figure 1A, these lncRNAs showed a general difference between P1 and P7. 32 lncRNAs exhibited a high fold change of at least 3-fold, where 9 lncRNAs exhibited increased expression and 23 lncRNAs decreased expression (Supplementary Material). The the 685 differentially expressed lncRNAs in mouse cardiac tissue were list in the supplementary materials.

From the analysis of sequencing data, 1833 differentially expressed mRNAs were found with comparisons between 1-day-old and 7-day-old cardiac tissue, among which 859 were upregulated mRNAs and 974 were downregulated mRNAs in 7-day-old cardiac tissue compared with tissue from 1-day-old mice. Their distinct expression patterns are also presented in the hierarchical clustering analysis shown in Figure 1B.

### Genomic location of differentially expressed lncRNAs

**Figure 2:**
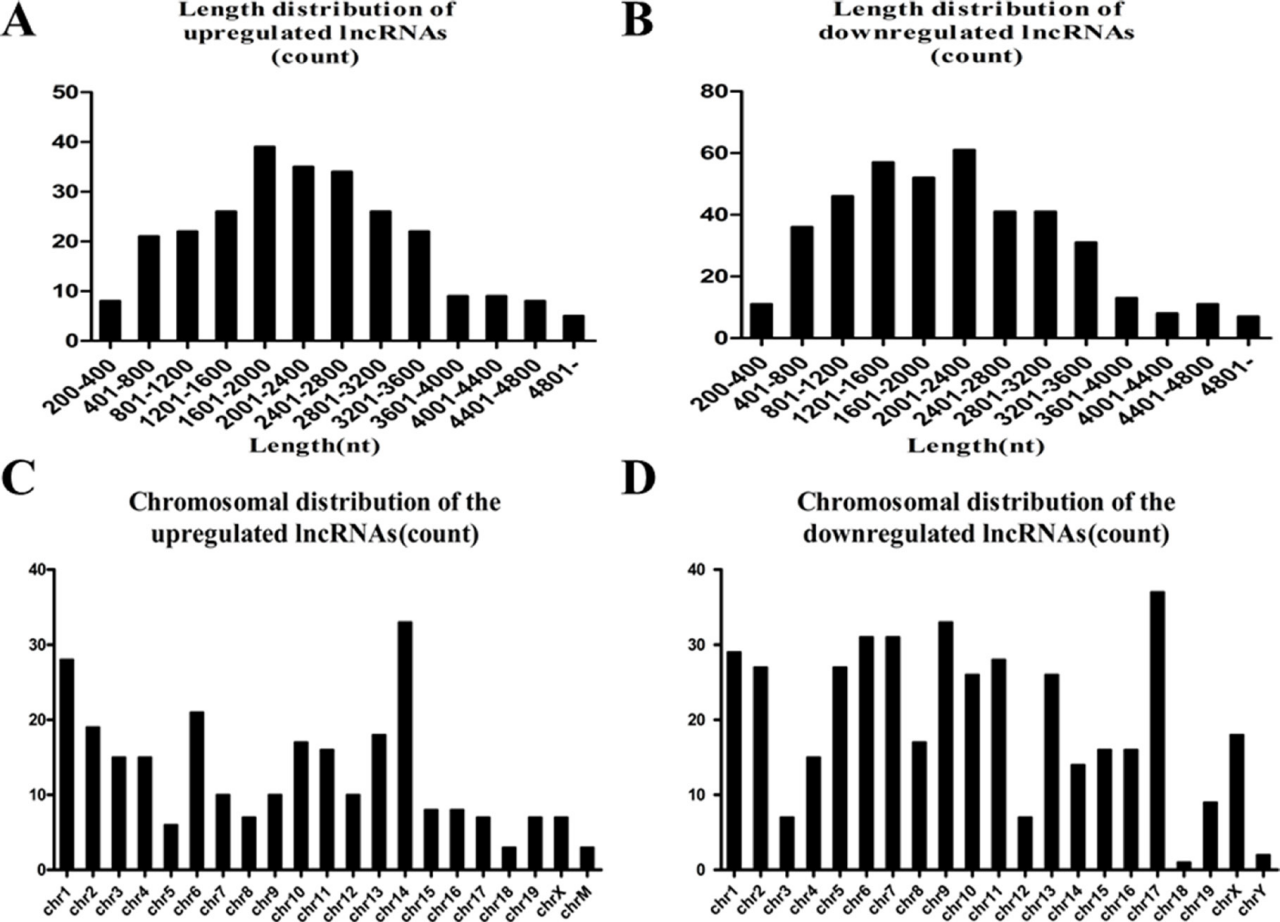
(**A**) Length distribution of the upregulated lncRNAs. (**B**) Length distribution of the downregulated lncRNAs. The upregulated and downregulated lncRNAs are mainly between 400 and 3600 bp in length. (**C**) Chromosome distribution shows the numbers of upregulated lncRNAs located in different chromosomes; chr represents chromosome. (**D**) The numbers of downregulated lncRNAs located in different chromosomes.

Length of the lncRNAs was generally between 400 bp and 3600 bp (Figure 2A, 2B). Chromosomal distribution showed the numbers of up- and downregulated lncRNAs located on different chromosomes (Figure 2C, 2D). Chromosomal distribution showed the upregulated lncRNAs were mainly located on chromosome 14, 1 and 6, and the downregulated lncRNAs were mainly located on chromosome 17, 9, 6 and 7. The functional characterisation of these lncRNAs presents a formidable task, and some studies suggest that lncRNAs regulate higher-order chromosomal dynamics, sub-cellular structural organisation and telomere biology [19]. LncRNAs have been suggested to originate from complex loci that include lncRNA and associated protein-coding genes, since several lncRNAs have been reported to regulate the expression of adjacent protein-coding genes [20, 21]. Such information about these lncRNAs and their nearby coding genes might be useful to predict their functional roles in heart regeneration (Table 1).

**Table 1:** lncRNAs and their nearby coding genes

Transcript	Upstream second gene	Upstream first gene	Downstream first gene	Downstream second gene
fantom3_B430306C15	fantom3_9230114P20	Slc10a6	1700016H13Rik	fantom3_F830205C23
NR_033497	fantom3_4930408C10	fantom3_A830009F23	Myh7	fantom3_D330040O14
fantom3_G630025L12	Kctd6	Acox2	fantom3_C630031G01	Fam107a
fantom3_D330013N04	fantom3_A330004D21	Myl4	Itgb3	fantom3_7030418D15
fantom3_B230377K03	Mt3	fantom3_A830083K16	Mt2	fantom3_1810074C23
fantom3_1110003P13	2700060E02Rik	fantom3_8030491K24	Gng2	fantom3_C130093P08
fantom3_4930408C10	Myh6	Mir208a	fantom3_A830009F23	D830015G02Rik
fantom3_9530030H05	Oxnad1	fantom3_2410002F01	Msmb	Ncoa4
NR_033813	fantom3_A330045P15	Begain	Meg3	fantom3_I0C0047O18
fantom3_2310031C01	Fam47e	fantom3_D530019K15	Stbd1	Ccdc158
fantom3_D530019K15	fantom3_C730043O17	Fam47e	fantom3_2310031C01	Stbd1
fantom3_2510038C20	Mt2	fantom3_1810074C23	Mt1	Nup93
fantom3_1810074C23	fantom3_B230377K03	Mt2	fantom3_2510038C20	Mt1
NR_030416	fantom3_I0C0030C13	fantom3_1100001A04	fantom3_D130045N19	Igf2
NR_001592	fantom3_9530074L01	fantom3_9830166K08	fantom3_I0C0030C13	fantom3_1100001A04
fantom3_I0C0030C13	fantom3_9830166K08	H19	fantom3_1100001A04	Mir675
fantom3_1100001A04	H19	fantom3_I0C0030C13	Mir675	fantom3_D130045N19
fantom3_9830143G06	Tmem164	fantom3_6030487A22	fantom3_4932428A02	fantom3_B230314K16
fantom3_4932428A02	fantom3_6030487A22	fantom3_9830143G06	fantom3_B230314K16	fantom3_C430013O12
fantom3_9230118C23	fantom3_D330009G10	2310050B05Rik	fantom3_2310050B05	Nmrk2
NR_015477	Dapk3	fantom3_D330009G10	fantom3_9230118C23	fantom3_2310050B05
fantom3_D330009G10	Snord37	Dapk3	2310050B05Rik	fantom3_9230118C23
fantom3_D130045N19	fantom3_1100001A04	Mir675	Igf2	Mir483
fantom3_1010001E19	fantom3_B230374I20	fantom3_B230350D20	fantom3_E430001N13	fantom3_D830012D22
fantom3_D830012D22	fantom3_1010001E19	fantom3_E430001N13	fantom3_0610011H19	fantom3_D030018G08
fantom3_E430001N13	fantom3_B230350D20	fantom3_1010001E19	fantom3_D830012D22	fantom3_0610011H19
fantom3_G730023M12	fantom3_A730057F09	Stc2	fantom3_F830230J18	Bod1
fantom3_4930578O05	fantom3_4930558J22	Hist1h4b	Hist1h4a	Hist1h3a
fantom3_A830073O21	Gm4971	fantom3_A730020G15	Fam174b	fantom3_4932443D20
fantom3_2310024F14	fantom3_A730026F12	Fxyd6	Fxyd2	fantom3_1700101G12
fantom3_A630066O12	Ank1	fantom3_6330529I17	Mir486	Mir3107
NR_039592	fantom3_E030018F15	Kctd12	fantom3_4933432I03	4933432I03Rik
fantom3_D630044L13	fantom3_F930015M13	Mir3966	fantom3_9530076L14	Dcn
fantom3_1700119I11	Zcchc9	Ckmt2	fantom3_6430599K20	fantom3_D830014B08
fantom3_C130052N24	Stard4	fantom3_E230006P11	Nrep	fantom3_D930015L03
fantom3_D830028G10	Lrtm1	fantom3_A930039P05	fantom3_D130049P09	fantom3_B930020O18
fantom3_A230064C13	Crhr1	Sppl2c	Mapt	Kansl1
fantom3_2210409C20	Itgb6	fantom3_E030019H01	fantom3_F420008A03	fantom3_I920190L12
fantom3_2310015A16	Sik1	fantom3_C130051M16	fantom3_C130017E03	Hsf2bp
fantom3_B230352I09	fantom3_C230086B22	fantom3_4933422O15	fantom3_2010321I05	Tnrc6a

### Validation of differentially expressed lncRNAs

**Figure 3:**
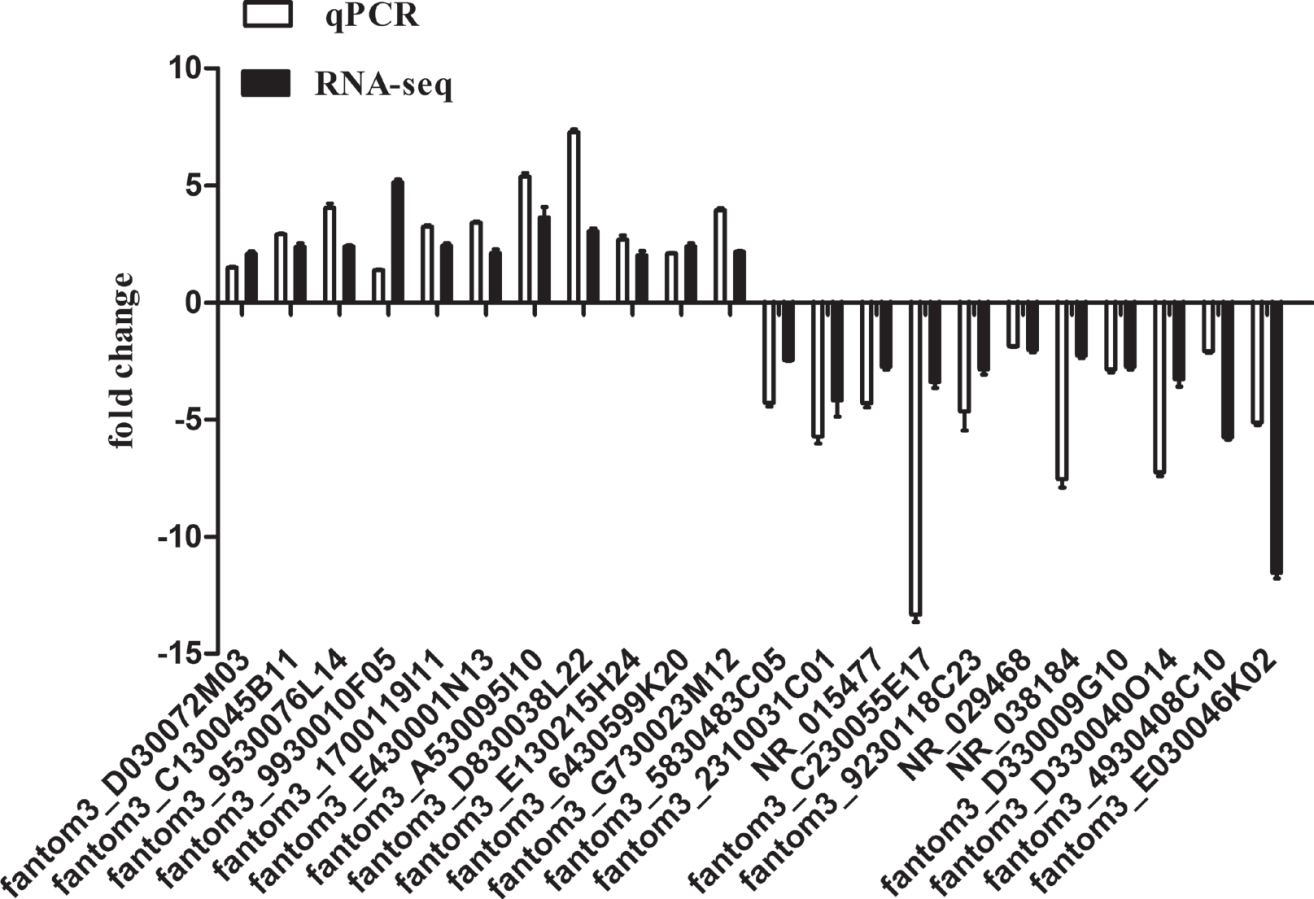
Comparison between sequencing data and the qRT-PCR result for lncRNAs Columns above the X-axis indicate the upregulated lncRNAs, and columns below the X-axis represent the downregulated lncRNAs.-PCR. The validation results indicated that the sequencing data correlated well with the qPCR results.

To verify the RNA-Seq data, we randomly selected 22 differentially expressed lncRNAs for qPCR analysis, among which 11 were upregulated lncRNAs and 11 were downregulated lncRNAs in 7-day-old cardiac tissue compared with tissue from 1-day-old mice. The result showed that the expression patterns of those lncRNAs were consistent with the RNA-Seq data (Figure 3).

### GO and pathway analysis

**Figure 4:**
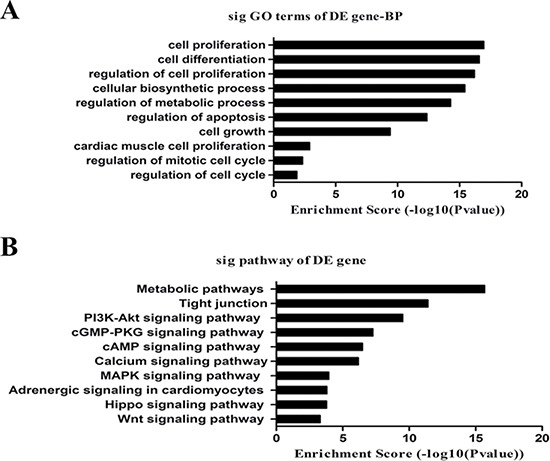
GO and pathway analysis (**A**) The list of the top 10 GO terms with the associated coding gene function of dysregulated lncRNAs. (**B**) The list of the top 10 pathways associated with the coding gene of dysregulated lncRNAs.

We performed GO analysis for the coding genes associated with differentially expressed lncRNAs as shown in Figure 4A. The top 10 terms associated with biological processes were: (1) GO:0008283: cell proliferation; (2) GO:0030154: cell differentiation; (3) GO:0042127: regulation of cell proliferation; (4) GO:0044249: cellular biosynthetic process; (5) GO:0019222: regulation of metabolism; (6) GO:0042981: regulation of apoptosis; (7) GO:0016049: cell growth; (8) GO:0060038: cardiac muscle cell proliferation; (9) GO:0007346: regulation of the mitotic cell cycle; and (10) GO:0051726: regulation of the cell cycle.

**Figure 5:**
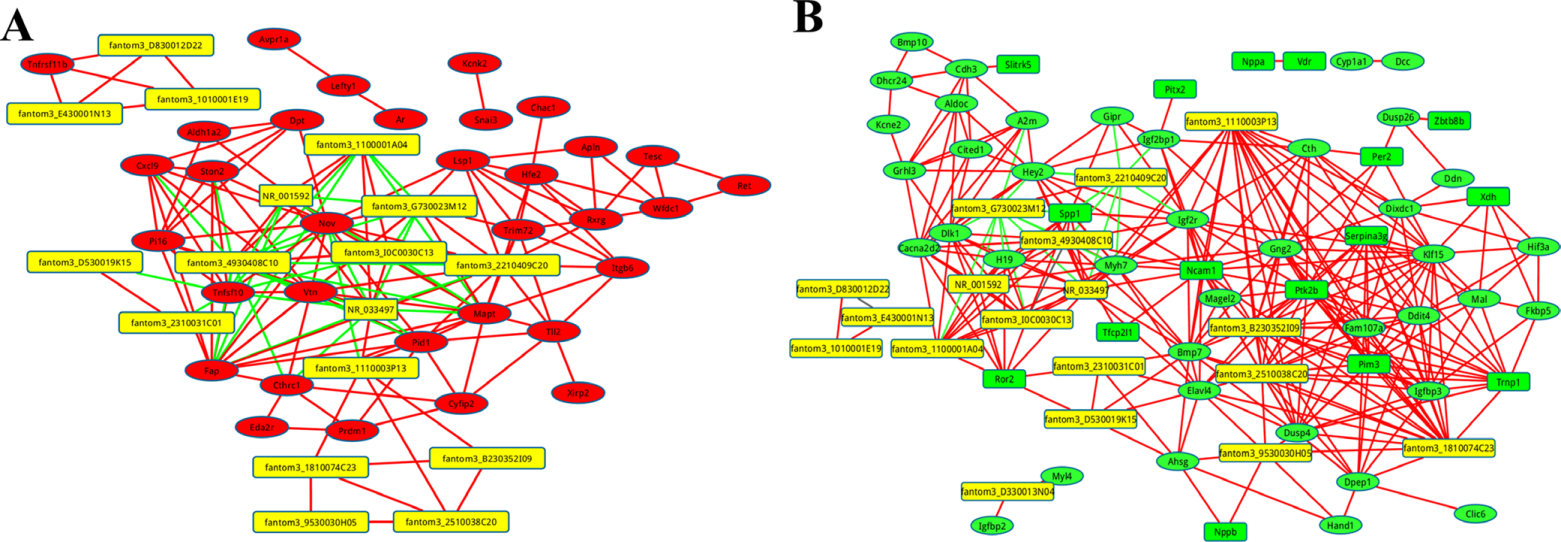
The co-expression network of the coding genes comparing P1 and P7, with the highly-dysregulated lncRNAs A correlation of > 0.99, or a correlation < −0.99 and *P* value of < 0.05 were recognised as co-expression. Red lines indicate a positive correlation, green lines indicate a negative correlation. (**A**) The co-expression network of the upregulated coding genes. A red circle indicates the upregulated coding genes, and a yellow square indicates lncRNAs. (**B**) The co-expression network of the downregulated coding genes. A green circle indicates the downregulated coding genes, a yellow square indicates LncRNAs.

We also performed KEGG pathway enrichment analysis for the coding genes associated with differentially expressed lncRNAs. The most significant pathways were: metabolic, tight junctions and signalling pathways including PI3K-Akt, cGMP-PKG, cAMP, MAPK, calcium, adrenergic signalling in cardiomyocytes, Hippo and Wnt (Figure 4B).

### CNC network

Biological networks cover modules of genes or proteins that may function in the same pathway; genes or proteins inside the same module can be co-regulated [22]. Therefore, construction of a CNC network could infer the biological functions of lncRNAs [22, 23], which would increase our understanding of the complex molecular mechanisms of heart regeneration.

Our data showed that the downregulated co-expression network was composed of 74 network nodes and 328 connections between 18 lncRNAs and 67 coding genes. The upregulated co-expression network was composed of 51 network nodes and 158 connections between 17 lncRNAs and 43 coding genes. In both of the co-expression networks, there was a positive correlation between most of the pairs. In addition, we observed that one lncRNA could target several coding genes and that one coding gene correlated with several lncRNAs. For example, in the downregulated co-expression network, the downregulated lncRNAs fantom3_B230352I09 and fantom3_1110003P13 were correlated with 17 and 15 coding genes respectively. In the upregulated co-expression network, lncRNA fantom3_2210409C20 and fantom3_G730023M12, upregulated in P7, were co-expressed with 8 and 7 coding genes respectively. By the coding-noncoding gene co-expression network, we identified some correlated targets of highly-dysregulated lncRNAs such as Igfbp3, Trnp1 Itgb6, and Pim3. As shown in Figure 5, those lncRNAs with the highest co-expression show the most important position.

## DISCUSSION

Although various miRNAs [24], transcription factors [25], growth factors [26], and cell cycle regulatory elements [27, 28] have been shown to regulate the genes that orchestrate proliferation during the regeneration of cardiomyocytes, the role of lncRNAs is poorly understood. Compared with adult mammals, neonatal mice have full capacity for cardiac regeneration after injury. Therefore, charting the transcriptional profile of lncRNAs in regenerative hearts from neonatal mice is a key step in understanding the role of lncRNAs, and this study was the first to examine the expression of lncRNAs in regenerative hearts.

Using next-generation high-throughput RNA sequencing techniques, this study found 685 lncRNAs that were differentially expressed in the heart between postnatal days 1 and 7 (P1 and P7, respectively), which consisted of 418 downregulated and 267 upregulated lncRNAs. In addition, 1833 differentially expressed mRNAs were identified, consisting of 859 upregulated and 974 downregulated mRNAs. 11 upregulated lncRNAs and 11 downregulated lncRNAs were further validated using qRT-PCR, and the results were consistent with the data of RNAseq. In addition, chromosomal distribution showed the upregulated lncRNAs were mainly located on chromosome 14, 1 and 6, and the downregulated lncRNAs were mainly located on chromosome 17, 9, 6 and 7. Interestingly, the Notch1 located on the chromosome 9 and TBX2 located on the chromosome 17 are closely related to heart development [29, 30].

We also used GO and pathway analyses to identify potential biological functions enriched among the associated genes of differentially expressed lncRNAs. GO functional enrichment analysis showed that the coding genes associated with differentially expressed lncRNAs were significantly involved in the following processes: cell proliferation and its regulation, cell differentiation, the cellular biosynthetic process; regulation of metabolism, regulation of apoptosis, cell growth, cardiac muscle cell proliferation and regulation of the mitotic and cell cycles. Stimulating endogenous cardiac regeneration by increasing the cell cycle activity of cardiomyocytes could increase the number of cardiomyocytes in an injured heart [31]. Previous studies have suggested that lncRNAs play a role in cell cycle regulation and cardiac development [32–34] and therefore that they are involved in cell cycle regulation of cardiomyocytes and cardiac regeneration [35]. Our study provides further evidence of the potential role of differentially expressed lncRNAs in the regenerative potential of heart tissue in neonatal mice, via regulation of the differentially expressed genes associated with differentiation and proliferation of cardiomyocytes.

Pathway-Express analysis identified that the metabolic pathways were most significantly affected by the coding genes associated with differentially expressed lncRNAs in 7-day-old cardiac tissue compared with tissue from 1-day-old mice. Macromolecules including glycogen, proteins, nucleotides and lipids represent both the cellular energy reserve and also the essential building blocks for cell renewal [36]. Neonatal mice retain the capacity for cardiac regeneration, but this is lost after 7 days. Therefore, the demand for the anabolic production of macromolecules in the regenerating heart increases during the first 6 days after birth.

The other significant signalling pathways were thePI3K-Akt, MAPK, Hippo, and adrenergic signalling in cardiomyocytes. Previous studies have shown that MAPK signalling pathways have non-redundant roles in the regulation of zebrafish cardiac regeneration [37]. The PI3K-AKT pathway promotes cardiomyocyte proliferation, survival, and physiological hypertrophy [38], and both the Hippo and Wnt signalling pathways also play an important role in cardiac regeneration [38–40]. In addition, it has been reported that sympathetic nerves have an important role in neonatal mammalian heart regeneration [41, 42]. Therefore, we hypothesised that those differentially expressed lncRNAs might participate in the process of neonatal cardiac regeneration by regulating the associated gene expression targeting these pathways.

To study the biological functions and potential mechanisms of differentially expressed lncRNAs, we constructed a CNC network on the basis of the correlation analysis between the differentially expressed lncRNAs and mRNA. Compared with the conventional differential expression analysis, the co-expression network analysis indicates the higher-order relationships of the transcripts in the biological framework [43]. In our co-expression network, we observed that some lncRNAs expression levels were markedly correlated with the expression of several protein-coding genes, indicating the importance of their location. For instance, the lncRNA fantom3_B230352I09, which was downregulated in P7, was co-expressed with 17 coding genes, most of which were related to apoptosis, cell growth, regulation of the cell cycle and cell proliferation. As another example, the Trnp1 gene [44] has been reported to accelerate cell cycle progression and the Pim3 gene [45] has a cardio-protective effect through the process of anti-apoptosis. Igfbp3 is a downstream target of Wnt signaling pathway and participates in the regulation of adult cardiac progenitor cell regeneration [46]. The lncRNA fantom3_2210409C20, upregulated in P7, was co-expressed with 8 coding genes. Among the co-expressed genes, Itgb6 (a β subunit of integrins) was found to be relatively close to fantom3_2210409C20. Integrins are expressed in all cellular compartments of the cardiovascular system and mediate cell adhesion, migration, proliferation and survival in many cell types [47]. Whether cardiac regeneration in 1-day-old neonatal mice is regulated by these lncRNAs warrants further study. Although our current understanding of lncRNA regulation in cardiac regeneration is in its infancy, several approaches can be employed to investigate their biological functions, including over-expression of lncRNA, lncRNA silencing and structure disruption, which may provide more conclusive evidence to explain the regulatory mechanisms.

In conclusion, we have shown the global different expression profiles of lncRNAs during the first seven days in the neonatal mouse heart. By bioinformatics prediction, we obtained some target genes of the candidate lncRNAs such as Igfbp3, Trnp1 Itgb6, and Pim3, which correlated with the process of proliferation. Collectively, our results suggest that lncRNAs could be important regulators in mammalian cardiac regeneration. Further work is needed to understand the biological functions and molecular mechanisms of specific lncRNAs implicated in cardiac regeneration.

## MATERIALS AND METHODS

### Animal and tissue preparation

The neonatal C57BL/6J mice were purchased from the Model Animal Research Centre of Nanjing University (Nanjing, China). Our study was approved by the Animal Care and Use Committee of Nanjing Medical University (Nanjing, China) and the methods were carried out in accordance with the approved guidelines. The left ventricular apex was removed from neonates 1 and 7 days after birth, cleaned and snap-frozen in liquid nitrogen. Three biological replicates were generated per timepoint. Hearts from five to ten mouse pups were pooled for each biological replicate.

### RNA extraction and quality control

Total RNA was extracted from neonatal mouse heart tissue using TRIzol reagent (Invitrogen Life Technologies) in accordance with the manufacturer's protocol. RNA quantity and quality were measured using a NanoDrop ND-1000. RNA integrity was assessed by standard denaturing agarose gel electrophoresis.

### Analysis of sequencing data

RNA-Seq based transcriptome profiling was performed by the Guangzhou RiboBio Co, using the high-throughput, high-sensitivity HiSeq 3000 sequencing platform (Illumina Company, USA). Clean data were acquired by removing joint sequence fragments, low-quality segments and ribosomal RNA. The short reads were mapped to the reference genome using TopHat [15] with 2 mismatches (−−read-mismatches = 2) and 2 gaps (read-gap-length = 2). Transcripts were assembled and the relative abundance of genes was estimated using Cufflinks in reads per kilobase of transcript per million mapped reads (RPKM) [16]. Differentially expressed lncRNAs between P1 and P7 were identified through fold change as well as *P* values calculated with a *t-test*. The threshold set for dysregulated lncRNAs was a fold change of ≥ 1.0 and a *P value* of ≤ 0.05. A hierarchical clustering analysis was applied to classify lncRNAs based on their expression levels.

### Quantitative PCR (qPCR) analysis

The results of RNA-Seq were validated via qPCR analysis. The cDNA was generated from 1 μg of total RNA using an AMV Reverse Transcriptase kit (Invitrogen Life Technologies). We designed primers for each lncRNA using Primer 3 (http://sourceforge.net/projects/primer3/) and used the Basic Local Alignment Search Tool from NCBI to verify that the amplified product was unique. The qPCR was performed using an ABI 7300 Sequence Detection system (Applied Biosystems, Foster City, CA, USA), under the following conditions: denaturation at 95°C for 10 min, and 40 cycles of 95°C for 15 sec and 60°C for 1 min. Relative gene expression levels were normalised to GAPDH and calculated according to the 2^−ΔCT^ method. Each sample was measured in triplicate and reproduced at least three times. The sequences of the primers used are shown in Table 2.

**Table 2:** PCR primer sequences used

Gene	Forward primer (5′-3′)	Reverse primer (3′-5′)
fantom3_E030046K02	CTGGAAGAGAGCACCAGACC	TGGTTTCTCACACCAGACCA
fantom3_4930408C10	TGGGGAGGAGAGAAAAGGTT	TGTGCCTCTCCCTTTCTGTT
fantom3_2310031C01	ACTCCCCAAGTGTGCTTTTG	TGTACAGTTCAAGGCTCTGTTTT
NR_015477	AGCGAGGCCAGCAGTCTAC	GGCCTCAGAATCCATGCTT
fantom3_C230055E17	AAGCCTCTATGGCAAGCGTA	AAGGCTGACCAAAAGCAGAA
fantom3_9230118C23	AGGTCCCAAGAGCACAGAGA	CAGGTATGAGGCCCTTGTGT
fantom3_5830483C05	TGCTGGTCACAAAAGTCAGG	GGTCGGTGGAGGAGTATGAA
NR_029468	GATTTTTCCGTCTGGTCCAA	CAGGCCGTCTTCTCAGACTC
NR_038184	CTATCCACCACACCCTGCTT	CCTTCTACATTCCCCACGAA
fantom3_D330009G10	AGCGAGGCCAGCAGTCTAC	GGCCTCAGAATCCATGCTT
fantom3_D330040O14	TAGCCAGAGTTGGTGTGCAG	AATGGCTTTGGTGTCTTTGG
fantom3_9930010F05	TATGCAATACCATGCCCAGA	CTTGGCTCCACTTCAAGAGC
fantom3_1700119I11	AGCATGGCTAACGGACAGAT	CCCATGATGCAAGATTTCCT
fantom3_E430001N13	GTACCGCAAGGGAAAGATGA	CCAGCTATCACCAAGCTCGT
fantom3_A530095I10	CCCTGCCCTGTAATTTTGAA	TATCCGGCTCTGCAAGAAGT
fantom3_D830038L22	GCTTTGCGTTTTCTGAGTCC	CATCCCTTTGCACTGTCCTT
fantom3_6430599K20	TTCCTTGGCTCCTAGCAGAA	CTATGCCCTTGTCTGGCAAT
fantom3_9530076L14	TGCCTACTTCTGCAACACCA	CCCACCCTCACCTCATAGTG
fantom3_G730023M12	CAAGTGCTGAAGGATGACCA	GCTGGAGAGGCTCAATCATC
fantom3_D030072M03	AACCAAGGCCAAGATTCCTT	GAGCCAGGCAATGAACAAAT
fantom3_E130215H24	AGGCAGCGCTCTGAGATAAG	CTGCGGGTGATTTATGTGTG
fantom3_C130045B11	GGCCTTCTGTTTGTGTGGTT	GCTTTCCACTCTCTGGCATC
GAPDH	ATTCAACGGCACAGTCAA	CTCGCTCCTGGAAGATGG

### GO and pathway analysis

Gene ontology (GO) analysis provides a controlled vocabulary to describe the molecular actions of gene products, their cellular locations and the biological processes in which those actions occur [17]. Fisher's exact test was used to detect any significant overlap between the differentially expressed list and the GO annotation list. The *P* value denotes the significance of GO term enrichment between differentially expressed genes (a *P* value ≤ 0.05 is accepted as significant). Pathway analysis was used to map genes to KEGG pathways. The *P* value (calculated via EASE-score, Fisher *P* value or hypergeometric *P* value) denotes the significance of the pathway correlations (a *P* value of ≤ 0.05 is recommended, since the smaller the *P* value, the greater the significance of the pathway).

### Construction of the coding-noncoding gene co-expression network

The coding-noncoding gene co-expression network (CNC network) was constructed based on the correlation analysis between the differentially expressed lncRNAs and mRNAs. The algorithm has been previously described [18]. Briefly, for each pair of genes analysed, we calculated the Pearson correlation coefficient (PCC) and correlation coefficient of the PCC between lncRNAs and coding genes (lncRNA-mRNA PCC and lncRNA-lncRNA PCC). We subsequently selected lncRNAs and mRNAs based on the PCC using the selection parameter PCC ≥ 0.99 as significant and created the co-expression networks using Cytoscape (Institute of Systems Biology, Seattle, US).

### Statistical analysis

The data were analysed using the SPSS 17.0 software package (SPSS, Chicago, IL, USA). Differential expression levels of lncRNAs were compared by independent-samples *t*-test between two groups, and Fisher's exact test was used in GO and pathway analysis. All values are expressed as the mean ± standard deviation from three independent experiments, and a *P* value of < 0.05 was considered statistically significant.

## SUPPLEMENTARY MATERIALS FIGURES AND TABLES




