# Supplementary material for: Identification of differentially expressed lncRNAs involved in transient regeneration of the neonatal C57BL/6J mouse heart by next-generation high-throughput RNA sequencing
Source: Oncotarget. 2017 Mar 3;8(17):28052–62. doi: 10.18632/oncotarget.15887 (PMC5438630; doi:10.18632/oncotarget.15887)
Supplement: Supplementary file 1 [file oncotarget-08-28052-s001.pdf]

www.impactjournals.com/oncotarget/ Oncotarget Supplementary Materials

Identification of differentially expressed lncRNAs involved in transient regeneration of the neonatal C57BL/6J mouse heart by next-generation high-throughput RNA sequencing

Supplementary Materials

Supplementary Table 1: The 685 differentially expressed lncRNAs in mouse cardiac tissue. See Supplementary_Table_1
