# Supplementary material for: Identification of differentially expressed lncRNAs involved in transient regeneration of the neonatal C57BL/6J mouse heart by next-generation high-throughput RNA sequencing
Source: Oncotarget. 2017 Mar 3;8(17):28052–62. doi: 10.18632/oncotarget.15887 (PMC5438630; doi:10.18632/oncotarget.15887)
Supplement: Supplementary file 2 [file oncotarget-08-28052-s002.docx]

**The 685 differentially expressed lncRNAs in mouse cardiac tissue**

|  | transcript | Gene | log2(Fold-change) | p-value |
| --- | --- | --- | --- | --- |
| 1 | fantom3_6030432O06 | fantom3_6030432O06 | -5.45447 | 1.54E-08 |
| 2 | fantom3_1200007C13 | fantom3_1200007C13 | -4.51277 | 1.35E-06 |
| 3 | fantom3_2900051M01 | fantom3_2900051M01 | -4.16054 | 1.35E-06 |
| 4 | fantom3_B430306C15 | fantom3_B430306C15 | -4.04525 | 1.82E-15 |
| 5 | fantom3_9630027E11 | fantom3_9630027E11 | -3.76949 | 1.69E-05 |
| 6 | fantom3_E330020H17 | fantom3_E330020H17 | -3.72045 | ######## |
| 7 | NR_033497 | D830015G02Rik | -3.68871 | 0 |
| 8 | fantom3_A130059H01 | fantom3_A130059H01 | -3.63399 | 1.32E-14 |
| 9 | fantom3_K230308N03 | fantom3_K230308N03 | -3.53652 | 0.00011 |
| 10 | fantom3_E030046K02 | fantom3_E030046K02 | -3.53149 | 3.02E-59 |
| 11 | fantom3_F630016E07 | fantom3_F630016E07 | -3.49305 | 1.37E-24 |
| 12 | fantom3_A530056I24 | fantom3_A530056I24 | -3.48425 | 2.12E-16 |
| 13 | fantom3_F730205J02 | fantom3_F730205J02 | -3.37958 | 0.000205 |
| 14 | fantom3_4833438D02 | fantom3_4833438D02 | -3.33928 | 1.13E-17 |
| 15 | fantom3_G630025L12 | fantom3_G630025L12 | -3.1909 | ######## |
| 16 | fantom3_F730002H19 | fantom3_F730002H19 | -3.18079 | 7.62E-14 |
| 17 | fantom3_F630110E18 | fantom3_F630110E18 | -3.15644 | 7.67E-13 |
| 18 | fantom3_C630031G01 | fantom3_C630031G01 | -3.10309 | 1.49E-09 |
| 19 | fantom3_F830015A02 | fantom3_F830015A02 | -3.10089 | 7.97E-06 |
| 20 | fantom3_E030029E24 | fantom3_E030029E24 | -3.08317 | 7.47E-27 |
| 21 | fantom3_D330013N04 | fantom3_D330013N04 | -3.053 | 0 |
| 22 | fantom3_B230377K03 | fantom3_B230377K03 | -3.00366 | ######## |
| 23 | NR_036631 | Zim3 | -2.97696 | 0.000179 |
| 24 | fantom3_C730047I11 | fantom3_C730047I11 | -2.97591 | 2.62E-09 |
| 25 | fantom3_6030433G20 | fantom3_6030433G20 | -2.96151 | 2.50E-05 |
| 26 | fantom3_C130072L14 | fantom3_C130072L14 | -2.95395 | 5.04E-15 |
| 27 | fantom3_D630002E24 | fantom3_D630002E24 | -2.81361 | 1.53E-06 |
| 28 | fantom3_B230209F15 | fantom3_B230209F15 | -2.8087 | 8.83E-07 |
| 29 | fantom3_I830059E05 | fantom3_I830059E05 | -2.79824 | 8.41E-40 |
| 30 | fantom3_5830408D10 | fantom3_5830408D10 | -2.76965 | 2.64E-06 |
| 31 | fantom3_4833404E15 | fantom3_4833404E15 | -2.74287 | 1.75E-10 |
| 32 | fantom3_4833404L02 | fantom3_4833404L02 | -2.74287 | 1.75E-10 |
| 33 | fantom3_D230018A04 | fantom3_D230018A04 | -2.64334 | 1.15E-09 |
| 34 | fantom3_8030491K24 | fantom3_8030491K24 | -2.63218 | 9.42E-60 |
| 35 | fantom3_2700078F24 | fantom3_2700078F24 | -2.62074 | 1.03E-56 |
| 36 | fantom3_1110003P13 | fantom3_1110003P13 | -2.61601 | ######## |
| 37 | NR_015605 | 2900052N01Rik | -2.5866 | 2.26E-05 |
| 38 | fantom3_C630003E24 | fantom3_C630003E24 | -2.5866 | 2.26E-05 |
| 39 | fantom3_I920077F18 | fantom3_I920077F18 | -2.57899 | 5.42E-08 |
| 40 | NR_075099 | Ahsg | -2.55625 | 4.79E-44 |
| 41 | fantom3_A230011A20 | fantom3_A230011A20 | -2.55198 | 5.58E-05 |
| 42 | fantom3_C130050K11 | fantom3_C130050K11 | -2.54756 | ######## |
| 43 | fantom3_4930408C10 | fantom3_4930408C10 | -2.52278 | 0 |
| 44 | fantom3_B230399N12 | fantom3_B230399N12 | -2.51169 | 1.76E-28 |
| 45 | fantom3_D830033G01 | fantom3_D830033G01 | -2.49117 | 9.99E-85 |
| 46 | fantom3_5330429I04 | fantom3_5330429I04 | -2.46519 | 3.04E-62 |
| 47 | fantom3_D030044A18 | fantom3_D030044A18 | -2.4629 | 2.02E-30 |
| 48 | fantom3_D630021F02 | fantom3_D630021F02 | -2.45878 | 1.58E-24 |
| 49 | fantom3_4732497D23 | fantom3_4732497D23 | -2.38878 | 2.22E-13 |
| 50 | fantom3_E330037B04 | fantom3_E330037B04 | -2.37434 | 2.26E-05 |
| 51 | fantom3_A830008I04 | fantom3_A830008I04 | -2.36895 | 2.56E-05 |
| 52 | fantom3_B930095M22 | fantom3_B930095M22 | -2.34505 | 1.88E-20 |
| 53 | fantom3_A430046E07 | fantom3_A430046E07 | -2.27277 | 6.10E-06 |
| 54 | fantom3_C130040H19 | fantom3_C130040H19 | -2.25919 | 1.08E-16 |
| 55 | fantom3_2210408O14 | fantom3_2210408O14 | -2.25441 | 2.99E-18 |
| 56 | fantom3_A730011L03 | fantom3_A730011L03 | -2.23423 | ######## |
| 57 | fantom3_A330086M03 | fantom3_A330086M03 | -2.2184 | 1.08E-05 |
| 58 | fantom3_E030042G24 | fantom3_E030042G24 | -2.21334 | 1.17E-60 |
| 59 | fantom3_D730001M18 | fantom3_D730001M18 | -2.21124 | 6.57E-07 |
| 60 | fantom3_9530030H05 | fantom3_9530030H05 | -2.20642 | 0 |
| 61 | fantom3_5830436I19 | fantom3_5830436I19 | -2.18836 | 5.90E-09 |
| 62 | fantom3_8030402F09 | fantom3_8030402F09 | -2.18778 | 1.45E-16 |
| 63 | fantom3_1110014L15 | fantom3_1110014L15 | -2.18143 | 6.34E-08 |
| 64 | fantom3_C130093P08 | fantom3_C130093P08 | -2.16964 | 0.000117 |
| 65 | fantom3_1700069H09 | fantom3_1700069H09 | -2.07602 | 9.99E-11 |
| 66 | NR_033813 | Dlk1 | -2.05208 | ######## |
| 67 | fantom3_8030469F12 | fantom3_8030469F12 | -2.03895 | 4.07E-10 |
| 68 | fantom3_9330178J21 | fantom3_9330178J21 | -2.02249 | 1.92E-34 |
| 69 | fantom3_A130027D14 | fantom3_A130027D14 | -2.01254 | 2.62E-13 |
| 70 | fantom3_2010204K13 | fantom3_2010204K13 | -1.97932 | 6.61E-08 |
| 71 | NR_027924 | 2010204K13Rik | -1.97931 | 6.61E-08 |
| 72 | fantom3_D730029L05 | fantom3_D730029L05 | -1.97339 | 2.60E-12 |
| 73 | fantom3_2310031C01 | fantom3_2310031C01 | -1.97086 | ######## |
| 74 | fantom3_D530019K15 | fantom3_D530019K15 | -1.96989 | ######## |
| 75 | fantom3_4932442M07 | fantom3_4932442M07 | -1.9552 | 1.05E-07 |
| 76 | fantom3_5330437E06 | fantom3_5330437E06 | -1.94581 | 6.61E-08 |
| 77 | fantom3_C230013K01 | fantom3_C230013K01 | -1.94439 | 4.67E-05 |
| 78 | fantom3_A230044F03 | fantom3_A230044F03 | -1.94276 | 4.67E-05 |
| 79 | fantom3_C920004M20 | fantom3_C920004M20 | -1.94245 | 1.13E-05 |
| 80 | NR_027925 | 2010204K13Rik | -1.93853 | 1.05E-07 |
| 81 | fantom3_7120498K14 | fantom3_7120498K14 | -1.9281 | 0.000196 |
| 82 | fantom3_2810458L13 | fantom3_2810458L13 | -1.9234 | 8.61E-25 |
| 83 | fantom3_D930045D13 | fantom3_D930045D13 | -1.92154 | 1.05E-07 |
| 84 | fantom3_1110005G03 | fantom3_1110005G03 | -1.92044 | 2.80E-05 |
| 85 | fantom3_2510038C20 | fantom3_2510038C20 | -1.90699 | 0 |
| 86 | fantom3_A130046O22 | fantom3_A130046O22 | -1.8926 | 1.53E-09 |
| 87 | fantom3_2900019G23 | fantom3_2900019G23 | -1.89175 | 2.10E-95 |
| 88 | fantom3_1810074C23 | fantom3_1810074C23 | -1.89152 | 0 |
| 89 | fantom3_6030426F17 | fantom3_6030426F17 | -1.88626 | 1.03E-07 |
| 90 | fantom3_A530041G03 | fantom3_A530041G03 | -1.88535 | 2.60E-07 |
| 91 | NR_024599 | Gm11346 | -1.8749 | 1.61E-08 |
| 92 | fantom3_D230037B08 | fantom3_D230037B08 | -1.87454 | 9.96E-08 |
| 93 | fantom3_C630002N23 | fantom3_C630002N23 | -1.86903 | 8.07E-24 |
| 94 | fantom3_6720436M04 | fantom3_6720436M04 | -1.86657 | 6.48E-14 |
| 95 | fantom3_C130041P16 | fantom3_C130041P16 | -1.8647 | 1.03E-08 |
| 96 | fantom3_6030498J18 | fantom3_6030498J18 | -1.8622 | 2.80E-05 |
| 97 | fantom3_E330030C20 | fantom3_E330030C20 | -1.85 | 6.88E-05 |
| 98 | fantom3_C130035O18 | fantom3_C130035O18 | -1.83751 | 4.57E-05 |
| 99 | fantom3_9630050E20 | fantom3_9630050E20 | -1.82719 | 9.95E-07 |
| 100 | fantom3_D130069E17 | fantom3_D130069E17 | -1.81851 | 2.80E-05 |
| 101 | fantom3_A130083M15 | fantom3_A130083M15 | -1.81245 | 2.52E-06 |
| 102 | NR_045477 | 1700003D09Rik | -1.80945 | 9.96E-08 |
| 103 | fantom3_1700003D09 | fantom3_1700003D09 | -1.80945 | 9.96E-08 |
| 104 | fantom3_D030040N13 | fantom3_D030040N13 | -1.79489 | 3.20E-09 |
| 105 | fantom3_6430501M24 | fantom3_6430501M24 | -1.79475 | 1.46E-10 |
| 106 | fantom3_E530001O09 | fantom3_E530001O09 | -1.78422 | 6.88E-05 |
| 107 | fantom3_9930022H22 | fantom3_9930022H22 | -1.78268 | 1.46E-06 |
| 108 | fantom3_C130032H02 | fantom3_C130032H02 | -1.77889 | 6.42E-06 |
| 109 | fantom3_A430075I01 | fantom3_A430075I01 | -1.77482 | 3.16E-14 |
| 110 | fantom3_C230055E17 | fantom3_C230055E17 | -1.76508 | ######## |
| 111 | fantom3_6820412L02 | fantom3_6820412L02 | -1.75894 | 3.11E-27 |
| 112 | fantom3_D130067J03 | fantom3_D130067J03 | -1.75802 | 1.13E-10 |
| 113 | fantom3_2610201A13 | fantom3_2610201A13 | -1.7518 | 3.75E-07 |
| 114 | fantom3_9430076L03 | fantom3_9430076L03 | -1.74814 | 4.43E-22 |
| 115 | fantom3_A630072N20 | fantom3_A630072N20 | -1.74454 | 1.41E-07 |
| 116 | fantom3_5330420E07 | fantom3_5330420E07 | -1.74026 | 9.58E-08 |
| 117 | fantom3_1110020M10 | fantom3_1110020M10 | -1.72454 | 2.33E-17 |
| 118 | fantom3_D330040O14 | fantom3_D330040O14 | -1.71077 | 8.97E-49 |
| 119 | fantom3_2900042E19 | fantom3_2900042E19 | -1.704 | 4.28E-48 |
| 120 | fantom3_B430214N02 | fantom3_B430214N02 | -1.69835 | 5.62E-16 |
| 121 | fantom3_2810025N20 | fantom3_2810025N20 | -1.66811 | 1.26E-22 |
| 122 | fantom3_F430003I07 | fantom3_F430003I07 | -1.66623 | 7.00E-09 |
| 123 | fantom3_9230106B05 | fantom3_9230106B05 | -1.6651 | 2.44E-08 |
| 124 | NR_030416 | Mir675 | -1.66182 | ######## |
| 125 | NR_001592 | H19 | -1.6605 | 0 |
| 126 | fantom3_4931431B13 | fantom3_4931431B13 | -1.65967 | 3.42E-10 |
| 127 | fantom3_I0C0030C13 | fantom3_I0C0030C13 | -1.65854 | 0 |
| 128 | fantom3_9330164E11 | fantom3_9330164E11 | -1.65509 | 9.79E-52 |
| 129 | fantom3_6430511F03 | fantom3_6430511F03 | -1.6538 | 1.07E-13 |
| 130 | fantom3_9430012O05 | fantom3_9430012O05 | -1.65093 | 8.38E-07 |
| 131 | fantom3_F830004L08 | fantom3_F830004L08 | -1.64751 | 2.10E-15 |
| 132 | fantom3_1100001A04 | fantom3_1100001A04 | -1.64379 | 0 |
| 133 | fantom3_A830028F24 | fantom3_A830028F24 | -1.63614 | 9.84E-10 |
| 134 | fantom3_3300001A09 | fantom3_3300001A09 | -1.62694 | 2.39E-20 |
| 135 | fantom3_E330003I04 | fantom3_E330003I04 | -1.62605 | 4.04E-07 |
| 136 | fantom3_2810047J09 | fantom3_2810047J09 | -1.62569 | 2.49E-06 |
| 137 | fantom3_A130004A03 | fantom3_A130004A03 | -1.61618 | 2.67E-07 |
| 138 | fantom3_9830143G06 | fantom3_9830143G06 | -1.6155 | 0 |
| 139 | fantom3_4932428A02 | fantom3_4932428A02 | -1.61532 | 0 |
| 140 | NR_027818 | 1500011B03Rik | -1.61216 | 9.11E-08 |
| 141 | fantom3_F930002H20 | fantom3_F930002H20 | -1.61144 | 5.11E-09 |
| 142 | fantom3_D130061L17 | fantom3_D130061L17 | -1.60855 | 9.47E-24 |
| 143 | fantom3_D830039K19 | fantom3_D830039K19 | -1.60438 | 1.23E-05 |
| 144 | NR_045295 | 2810029C07Rik | -1.60036 | 1.08E-07 |
| 145 | fantom3_7230402M02 | fantom3_7230402M02 | -1.5946 | 5.31E-16 |
| 146 | fantom3_4631402A18 | fantom3_4631402A18 | -1.59279 | 1.56E-53 |
| 147 | fantom3_1110004J10 | fantom3_1110004J10 | -1.59252 | ######## |
| 148 | fantom3_6030402G09 | fantom3_6030402G09 | -1.58966 | 1.74E-15 |
| 149 | NR_029465 | Gm11149 | -1.58741 | 3.00E-50 |
| 150 | fantom3_4930533K04 | fantom3_4930533K04 | -1.58741 | 3.00E-50 |
| 151 | fantom3_5830461L22 | fantom3_5830461L22 | -1.58739 | 1.85E-05 |
| 152 | fantom3_4732480P20 | fantom3_4732480P20 | -1.58619 | 3.21E-12 |
| 153 | fantom3_D430035A06 | fantom3_D430035A06 | -1.58313 | 4.64E-05 |
| 154 | fantom3_B020014I18 | fantom3_B020014I18 | -1.57101 | 4.45E-18 |
| 155 | fantom3_9630050M23 | fantom3_9630050M23 | -1.56488 | 1.53E-05 |
| 156 | fantom3_C130071M15 | fantom3_C130071M15 | -1.56087 | 4.62E-08 |
| 157 | fantom3_C030002P19 | fantom3_C030002P19 | -1.554 | 3.44E-08 |
| 158 | fantom3_E430012J02 | fantom3_E430012J02 | -1.55387 | 9.58E-19 |
| 159 | fantom3_A930009H07 | fantom3_A930009H07 | -1.55195 | 2.81E-27 |
| 160 | NR_045954 | 4833412C05Rik | -1.55185 | 2.56E-08 |
| 161 | fantom3_E430032J10 | fantom3_E430032J10 | -1.54918 | 0.000174 |
| 162 | fantom3_A130019J18 | fantom3_A130019J18 | -1.54877 | 3.07E-07 |
| 163 | fantom3_4833412C05 | fantom3_4833412C05 | -1.54469 | 6.90E-08 |
| 164 | fantom3_A330045H03 | fantom3_A330045H03 | -1.54132 | 7.52E-07 |
| 165 | fantom3_F730320N24 | fantom3_F730320N24 | -1.54079 | 1.24E-22 |
| 166 | fantom3_7420436A12 | fantom3_7420436A12 | -1.53599 | ######## |
| 167 | fantom3_4833432E10 | fantom3_4833432E10 | -1.53359 | 2.26E-06 |
| 168 | fantom3_D230005H13 | fantom3_D230005H13 | -1.52821 | 3.78E-05 |
| 169 | fantom3_A630081P21 | fantom3_A630081P21 | -1.52711 | 0.000104 |
| 170 | fantom3_A430024L20 | fantom3_A430024L20 | -1.52326 | 1.32E-17 |
| 171 | fantom3_C330006P03 | fantom3_C330006P03 | -1.52321 | 2.97E-19 |
| 172 | fantom3_9530014B21 | fantom3_9530014B21 | -1.52045 | 1.16E-68 |
| 173 | fantom3_9230114I18 | fantom3_9230114I18 | -1.51772 | 7.04E-59 |
| 174 | fantom3_9630025N01 | fantom3_9630025N01 | -1.51068 | 3.26E-14 |
| 175 | fantom3_A130066K21 | fantom3_A130066K21 | -1.49967 | 0.000214 |
| 176 | NR_102306 | D830032E09Rik | -1.49908 | 5.08E-09 |
| 177 | fantom3_E130309P10 | fantom3_E130309P10 | -1.49692 | 6.68E-06 |
| 178 | fantom3_D830027F07 | fantom3_D830027F07 | -1.49495 | 5.08E-09 |
| 179 | fantom3_E030015P15 | fantom3_E030015P15 | -1.49395 | 1.66E-06 |
| 180 | fantom3_C130048K06 | fantom3_C130048K06 | -1.49293 | 3.61E-06 |
| 181 | fantom3_D430036M17 | fantom3_D430036M17 | -1.49185 | 1.24E-06 |
| 182 | fantom3_D830032E09 | fantom3_D830032E09 | -1.48948 | 1.16E-09 |
| 183 | fantom3_9230118C23 | fantom3_9230118C23 | -1.48881 | 0 |
| 184 | fantom3_D330038P10 | fantom3_D330038P10 | -1.4875 | 1.19E-09 |
| 185 | fantom3_6330420E11 | fantom3_6330420E11 | -1.47774 | 2.03E-07 |
| 186 | fantom3_9430046L11 | fantom3_9430046L11 | -1.47322 | 5.96E-06 |
| 187 | NR_102307 | D830032E09Rik | -1.47022 | 2.21E-09 |
| 188 | fantom3_2010203O03 | fantom3_2010203O03 | -1.46963 | 3.31E-14 |
| 189 | fantom3_2610020E23 | fantom3_2610020E23 | -1.46963 | 3.31E-14 |
| 190 | fantom3_C530050E15 | fantom3_C530050E15 | -1.46616 | 8.15E-13 |
| 191 | fantom3_A430009E06 | fantom3_A430009E06 | -1.46567 | 3.02E-05 |
| 192 | fantom3_3930401B19 | fantom3_3930401B19 | -1.46289 | ######## |
| 193 | fantom3_D030011I02 | fantom3_D030011I02 | -1.45812 | 9.27E-10 |
| 194 | fantom3_1200016E24 | fantom3_1200016E24 | -1.45739 | ######## |
| 195 | fantom3_2810029C07 | fantom3_2810029C07 | -1.44746 | 4.45E-05 |
| 196 | fantom3_D130079M20 | fantom3_D130079M20 | -1.44714 | 2.38E-25 |
| 197 | NR_015477 | 2310050B05Rik | -1.44476 | ######## |
| 198 | fantom3_D330009G10 | fantom3_D330009G10 | -1.44476 | ######## |
| 199 | NR_030701 | Gm20554 | -1.44047 | 6.02E-08 |
| 200 | fantom3_C230086F11 | fantom3_C230086F11 | -1.43396 | 1.74E-06 |
| 201 | fantom3_E430013J17 | fantom3_E430013J17 | -1.43145 | 1.65E-25 |
| 202 | fantom3_A130027A01 | fantom3_A130027A01 | -1.43065 | 3.06E-18 |
| 203 | fantom3_E130116O09 | fantom3_E130116O09 | -1.42838 | 1.30E-10 |
| 204 | fantom3_9430069G01 | fantom3_9430069G01 | -1.42209 | 3.46E-09 |
| 205 | fantom3_A530026G04 | fantom3_A530026G04 | -1.41923 | ######## |
| 206 | fantom3_D130071D02 | fantom3_D130071D02 | -1.41507 | 8.75E-06 |
| 207 | fantom3_4833405D16 | fantom3_4833405D16 | -1.41353 | 2.21E-19 |
| 208 | fantom3_C730018L08 | fantom3_C730018L08 | -1.41162 | 0.000204 |
| 209 | fantom3_9330186K03 | fantom3_9330186K03 | -1.39677 | 1.64E-05 |
| 210 | fantom3_A130014B07 | fantom3_A130014B07 | -1.39388 | 4.99E-13 |
| 211 | NR_027820 | 1810032O08Rik | -1.38875 | 1.33E-16 |
| 212 | fantom3_2900054A12 | fantom3_2900054A12 | -1.38689 | 1.90E-16 |
| 213 | fantom3_9330160I12 | fantom3_9330160I12 | -1.38447 | 3.20E-34 |
| 214 | NR_015615 | 4732416N19Rik | -1.38019 | 1.02E-18 |
| 215 | fantom3_C130051K14 | fantom3_C130051K14 | -1.37902 | 4.09E-08 |
| 216 | fantom3_D030020C12 | fantom3_D030020C12 | -1.37491 | 1.30E-07 |
| 217 | fantom3_5430408K11 | fantom3_5430408K11 | -1.3738 | 8.63E-09 |
| 218 | fantom3_E130111P11 | fantom3_E130111P11 | -1.37149 | 1.10E-82 |
| 219 | NR_039574 | Mir5114 | -1.36717 | 5.14E-19 |
| 220 | fantom3_4833407D20 | fantom3_4833407D20 | -1.36583 | 1.39E-81 |
| 221 | fantom3_6030440J16 | fantom3_6030440J16 | -1.35479 | 6.24E-42 |
| 222 | NR_045354 | 4930483K19Rik | -1.35378 | 0.000202 |
| 223 | fantom3_4930483K19 | fantom3_4930483K19 | -1.35378 | 0.000202 |
| 224 | fantom3_1190007F10 | fantom3_1190007F10 | -1.34826 | 2.78E-06 |
| 225 | fantom3_1110020B05 | fantom3_1110020B05 | -1.34669 | ######## |
| 226 | fantom3_D430040C13 | fantom3_D430040C13 | -1.34334 | 6.54E-05 |
| 227 | fantom3_6720429I20 | fantom3_6720429I20 | -1.34163 | ######## |
| 228 | fantom3_6230417F24 | fantom3_6230417F24 | -1.33864 | 4.14E-13 |
| 229 | fantom3_C130053B21 | fantom3_C130053B21 | -1.33773 | 1.54E-06 |
| 230 | fantom3_1200003N03 | fantom3_1200003N03 | -1.33621 | 0 |
| 231 | fantom3_B130017A18 | fantom3_B130017A18 | -1.33486 | 9.50E-53 |
| 232 | fantom3_A130084B22 | fantom3_A130084B22 | -1.33366 | 4.17E-06 |
| 233 | fantom3_7120485G19 | fantom3_7120485G19 | -1.33271 | 2.14E-52 |
| 234 | fantom3_5830403L16 | fantom3_5830403L16 | -1.33245 | ######## |
| 235 | fantom3_6720469I21 | fantom3_6720469I21 | -1.3274 | 5.45E-12 |
| 236 | fantom3_E030020H05 | fantom3_E030020H05 | -1.3265 | 5.73E-19 |
| 237 | fantom3_4930566C02 | fantom3_4930566C02 | -1.32479 | 7.04E-55 |
| 238 | fantom3_1110002G11 | fantom3_1110002G11 | -1.31718 | 1.47E-84 |
| 239 | fantom3_A630063J07 | fantom3_A630063J07 | -1.31544 | 3.02E-09 |
| 240 | fantom3_6430515G23 | fantom3_6430515G23 | -1.3154 | 6.24E-14 |
| 241 | fantom3_A430078A03 | fantom3_A430078A03 | -1.31508 | 2.84E-81 |
| 242 | fantom3_D330048J03 | fantom3_D330048J03 | -1.31466 | 3.59E-09 |
| 243 | fantom3_A530048O11 | fantom3_A530048O11 | -1.31349 | 4.00E-62 |
| 244 | fantom3_D830016K24 | fantom3_D830016K24 | -1.31287 | 3.86E-18 |
| 245 | fantom3_5430417L22 | fantom3_5430417L22 | -1.31138 | 4.01E-64 |
| 246 | fantom3_9230006B08 | fantom3_9230006B08 | -1.3082 | 0.000101 |
| 247 | fantom3_9930032F22 | fantom3_9930032F22 | -1.30685 | 1.89E-10 |
| 248 | fantom3_4833421B09 | fantom3_4833421B09 | -1.30386 | 4.13E-06 |
| 249 | fantom3_D930020N11 | fantom3_D930020N11 | -1.29906 | 3.84E-64 |
| 250 | fantom3_2310010G08 | fantom3_2310010G08 | -1.29905 | 8.92E-28 |
| 251 | fantom3_5830483C05 | fantom3_5830483C05 | -1.29416 | 6.45E-43 |
| 252 | fantom3_D930025F21 | fantom3_D930025F21 | -1.29073 | 0.000122 |
| 253 | fantom3_E030017G24 | fantom3_E030017G24 | -1.28795 | 7.62E-58 |
| 254 | NR_027819 | 1810032O08Rik | -1.2869 | 5.25E-20 |
| 255 | fantom3_5830447G06 | fantom3_5830447G06 | -1.28241 | 5.35E-05 |
| 256 | fantom3_G930026B02 | fantom3_G930026B02 | -1.28143 | 4.03E-05 |
| 257 | NR_002855 | Igf2as | -1.28068 | 2.22E-36 |
| 258 | fantom3_2310067L22 | fantom3_2310067L22 | -1.27955 | ######## |
| 259 | fantom3_F830220H05 | fantom3_F830220H05 | -1.27794 | 2.44E-06 |
| 260 | fantom3_E430024C06 | fantom3_E430024C06 | -1.27672 | 5.90E-59 |
| 261 | fantom3_A130085K02 | fantom3_A130085K02 | -1.27278 | 4.94E-06 |
| 262 | NR_046017 | 9530052E02Rik | -1.2699 | 2.76E-10 |
| 263 | fantom3_9530052E02 | fantom3_9530052E02 | -1.2699 | 2.76E-10 |
| 264 | fantom3_6430408O03 | fantom3_6430408O03 | -1.26761 | 0.00022 |
| 265 | fantom3_5730409N24 | fantom3_5730409N24 | -1.26318 | 4.55E-06 |
| 266 | fantom3_E430016H06 | fantom3_E430016H06 | -1.26309 | 2.38E-55 |
| 267 | NR_030716 | 5430417L22Rik | -1.26016 | ######## |
| 268 | fantom3_B830031E22 | fantom3_B830031E22 | -1.25955 | 1.17E-14 |
| 269 | fantom3_E030041H03 | fantom3_E030041H03 | -1.25918 | 6.62E-11 |
| 270 | fantom3_F630221E23 | fantom3_F630221E23 | -1.25513 | 1.72E-10 |
| 271 | fantom3_9430077P08 | fantom3_9430077P08 | -1.25249 | 1.89E-18 |
| 272 | fantom3_4732410D20 | fantom3_4732410D20 | -1.24785 | 1.86E-06 |
| 273 | fantom3_4832418E19 | fantom3_4832418E19 | -1.24736 | 9.35E-14 |
| 274 | fantom3_2610103A09 | fantom3_2610103A09 | -1.2418 | 1.59E-28 |
| 275 | fantom3_A330054L18 | fantom3_A330054L18 | -1.23852 | 4.33E-12 |
| 276 | fantom3_E130311L23 | fantom3_E130311L23 | -1.23625 | 1.25E-07 |
| 277 | fantom3_2410024N18 | fantom3_2410024N18 | -1.23256 | 9.84E-11 |
| 278 | fantom3_A230062J21 | fantom3_A230062J21 | -1.22897 | 7.28E-14 |
| 279 | fantom3_6030408B16 | fantom3_6030408B16 | -1.22568 | 1.81E-10 |
| 280 | fantom3_6330403I03 | fantom3_6330403I03 | -1.22538 | ######## |
| 281 | fantom3_F630113A11 | fantom3_F630113A11 | -1.22504 | 5.48E-08 |
| 282 | fantom3_A730094I17 | fantom3_A730094I17 | -1.22465 | 3.26E-13 |
| 283 | NR_040280 | Gm12295 | -1.2238 | 1.05E-16 |
| 284 | fantom3_A730013G04 | fantom3_A730013G04 | -1.2238 | 1.05E-16 |
| 285 | fantom3_4930588G17 | fantom3_4930588G17 | -1.22314 | 3.08E-07 |
| 286 | fantom3_4933431K23 | fantom3_4933431K23 | -1.22016 | 3.30E-05 |
| 287 | fantom3_A130056N22 | fantom3_A130056N22 | -1.21939 | 5.57E-12 |
| 288 | fantom3_6820427D17 | fantom3_6820427D17 | -1.21577 | 1.06E-43 |
| 289 | fantom3_7030405B14 | fantom3_7030405B14 | -1.21298 | 1.01E-62 |
| 290 | fantom3_E130110N23 | fantom3_E130110N23 | -1.20998 | 1.38E-23 |
| 291 | fantom3_D830013E14 | fantom3_D830013E14 | -1.20141 | 9.20E-38 |
| 292 | fantom3_A130010C23 | fantom3_A130010C23 | -1.1998 | 1.21E-05 |
| 293 | fantom3_A430018G11 | fantom3_A430018G11 | -1.19969 | 2.76E-14 |
| 294 | fantom3_6430510N09 | fantom3_6430510N09 | -1.18991 | 1.41E-43 |
| 295 | fantom3_B230207G04 | fantom3_B230207G04 | -1.18921 | 3.98E-07 |
| 296 | fantom3_C820006D16 | fantom3_C820006D16 | -1.1888 | 7.47E-16 |
| 297 | fantom3_D830028M12 | fantom3_D830028M12 | -1.18695 | 5.69E-05 |
| 298 | NR_038184 | C430049B03Rik | -1.18679 | ######## |
| 299 | fantom3_2600014K08 | fantom3_2600014K08 | -1.18505 | 3.24E-17 |
| 300 | fantom3_9830123H11 | fantom3_9830123H11 | -1.18388 | 2.62E-13 |
| 301 | NR_038185 | C430049B03Rik | -1.18109 | 4.92E-97 |
| 302 | fantom3_A630071G03 | fantom3_A630071G03 | -1.1779 | 4.63E-66 |
| 303 | fantom3_9330107J05 | fantom3_9330107J05 | -1.17474 | 3.94E-05 |
| 304 | fantom3_2900016L01 | fantom3_2900016L01 | -1.17269 | 4.14E-09 |
| 305 | NR_073190 | Chka | -1.17218 | 3.20E-51 |
| 306 | fantom3_G630022B10 | fantom3_G630022B10 | -1.17152 | 3.12E-06 |
| 307 | fantom3_D130045N19 | fantom3_D130045N19 | -1.1706 | 0 |
| 308 | fantom3_E430003N15 | fantom3_E430003N15 | -1.16797 | 1.03E-13 |
| 309 | fantom3_9330131L20 | fantom3_9330131L20 | -1.16725 | 2.26E-05 |
| 310 | fantom3_2810490L08 | fantom3_2810490L08 | -1.16631 | 2.84E-46 |
| 311 | fantom3_D030073H18 | fantom3_D030073H18 | -1.1657 | 1.62E-10 |
| 312 | fantom3_C130030N11 | fantom3_C130030N11 | -1.16124 | 4.95E-24 |
| 313 | fantom3_D930047M05 | fantom3_D930047M05 | -1.15988 | 4.78E-23 |
| 314 | fantom3_4930558E18 | fantom3_4930558E18 | -1.15953 | 2.01E-06 |
| 315 | fantom3_B230303K20 | fantom3_B230303K20 | -1.15947 | 5.65E-37 |
| 316 | fantom3_C130030E20 | fantom3_C130030E20 | -1.1578 | 2.11E-19 |
| 317 | fantom3_A130029O08 | fantom3_A130029O08 | -1.15733 | 2.21E-16 |
| 318 | fantom3_0610023I12 | fantom3_0610023I12 | -1.1565 | 2.84E-08 |
| 319 | fantom3_9030407P20 | fantom3_9030407P20 | -1.15641 | 8.52E-13 |
| 320 | fantom3_D330035F22 | fantom3_D330035F22 | -1.15542 | 8.06E-07 |
| 321 | fantom3_D830007B15 | fantom3_D830007B15 | -1.154 | 2.28E-09 |
| 322 | fantom3_C630001G20 | fantom3_C630001G20 | -1.1537 | 7.19E-16 |
| 323 | fantom3_5430431A17 | fantom3_5430431A17 | -1.15131 | 3.24E-25 |
| 324 | fantom3_6430502G17 | fantom3_6430502G17 | -1.14808 | 0.000157 |
| 325 | fantom3_E030004N14 | fantom3_E030004N14 | -1.14681 | 4.77E-33 |
| 326 | fantom3_8030428J02 | fantom3_8030428J02 | -1.14493 | 2.17E-09 |
| 327 | fantom3_9530026L11 | fantom3_9530026L11 | -1.14346 | 0.000223 |
| 328 | fantom3_9330140P17 | fantom3_9330140P17 | -1.14315 | 9.23E-13 |
| 329 | fantom3_A130040F22 | fantom3_A130040F22 | -1.14124 | 0.000183 |
| 330 | fantom3_5430417G01 | fantom3_5430417G01 | -1.14049 | 1.22E-16 |
| 331 | fantom3_5430434E17 | fantom3_5430434E17 | -1.13693 | 1.21E-19 |
| 332 | fantom3_9230110L19 | fantom3_9230110L19 | -1.1369 | 5.32E-05 |
| 333 | NR_033803 | 6030408B16Rik | -1.13361 | 2.47E-10 |
| 334 | fantom3_8030487C13 | fantom3_8030487C13 | -1.13101 | 3.68E-20 |
| 335 | NR_027821 | 1810032O08Rik | -1.13024 | 3.10E-21 |
| 336 | fantom3_A730055O20 | fantom3_A730055O20 | -1.12739 | 2.10E-15 |
| 337 | fantom3_A630039H14 | fantom3_A630039H14 | -1.12409 | 1.85E-49 |
| 338 | fantom3_5730408B21 | fantom3_5730408B21 | -1.12322 | 1.46E-15 |
| 339 | fantom3_D330015C18 | fantom3_D330015C18 | -1.12175 | 3.67E-49 |
| 340 | fantom3_E130014G18 | fantom3_E130014G18 | -1.11901 | 5.71E-22 |
| 341 | fantom3_A630054M19 | fantom3_A630054M19 | -1.11791 | 5.02E-09 |
| 342 | fantom3_G730026I11 | fantom3_G730026I11 | -1.11268 | 3.84E-34 |
| 343 | NR_040401 | C920006O11Rik | -1.10948 | 3.44E-06 |
| 344 | fantom3_C920006O11 | fantom3_C920006O11 | -1.10948 | 3.44E-06 |
| 345 | fantom3_9130232E19 | fantom3_9130232E19 | -1.10777 | 4.59E-06 |
| 346 | fantom3_4732457O16 | fantom3_4732457O16 | -1.10646 | 7.49E-05 |
| 347 | fantom3_9430092O13 | fantom3_9430092O13 | -1.10499 | 5.42E-06 |
| 348 | fantom3_D930008O12 | fantom3_D930008O12 | -1.10449 | 0 |
| 349 | fantom3_6430545C09 | fantom3_6430545C09 | -1.10108 | 1.06E-08 |
| 350 | fantom3_E130105D15 | fantom3_E130105D15 | -1.10066 | 3.28E-66 |
| 351 | fantom3_8430417O13 | fantom3_8430417O13 | -1.09833 | 7.43E-06 |
| 352 | fantom3_D330018A11 | fantom3_D330018A11 | -1.09324 | 4.08E-07 |
| 353 | fantom3_F630107H02 | fantom3_F630107H02 | -1.09267 | 9.29E-09 |
| 354 | fantom3_C130031A05 | fantom3_C130031A05 | -1.09173 | 0.000164 |
| 355 | fantom3_A630091J10 | fantom3_A630091J10 | -1.09125 | 2.53E-07 |
| 356 | fantom3_A630095M19 | fantom3_A630095M19 | -1.08904 | 5.32E-05 |
| 357 | fantom3_D230035C20 | fantom3_D230035C20 | -1.08566 | 6.66E-08 |
| 358 | fantom3_A330051H13 | fantom3_A330051H13 | -1.08517 | ######## |
| 359 | fantom3_2310051E17 | fantom3_2310051E17 | -1.08496 | 8.86E-29 |
| 360 | fantom3_5830485B09 | fantom3_5830485B09 | -1.08397 | 0.00012 |
| 361 | fantom3_2210018M03 | fantom3_2210018M03 | -1.08342 | 3.13E-08 |
| 362 | fantom3_A830025B07 | fantom3_A830025B07 | -1.0832 | 2.60E-07 |
| 363 | fantom3_D830010O06 | fantom3_D830010O06 | -1.08268 | 7.2924089326168e-321 |
| 364 | fantom3_E030050E14 | fantom3_E030050E14 | -1.08098 | 1.01E-05 |
| 365 | NR_027664 | Adhfe1 | -1.0783 | 1.88E-70 |
| 366 | fantom3_3200004D15 | fantom3_3200004D15 | -1.07739 | 6.66E-08 |
| 367 | fantom3_9330209J16 | fantom3_9330209J16 | -1.07702 | 4.78E-07 |
| 368 | fantom3_E230025E14 | fantom3_E230025E14 | -1.07582 | 8.39E-10 |
| 369 | fantom3_4931417G19 | fantom3_4931417G19 | -1.0752 | 4.11E-16 |
| 370 | fantom3_C730043J07 | fantom3_C730043J07 | -1.07329 | 2.31E-08 |
| 371 | fantom3_C730045O03 | fantom3_C730045O03 | -1.07329 | 2.31E-08 |
| 372 | fantom3_A530076C18 | fantom3_A530076C18 | -1.07314 | 2.21E-07 |
| 373 | fantom3_A930011D19 | fantom3_A930011D19 | -1.07213 | 5.95E-12 |
| 374 | fantom3_D330046A10 | fantom3_D330046A10 | -1.06923 | 2.26E-08 |
| 375 | fantom3_5430439G14 | fantom3_5430439G14 | -1.0676 | 2.07E-09 |
| 376 | fantom3_D030011M09 | fantom3_D030011M09 | -1.06755 | 0.00012 |
| 377 | fantom3_B230343E22 | fantom3_B230343E22 | -1.06725 | 3.15E-18 |
| 378 | NR_028540 | Snord12 | -1.06469 | 5.50E-06 |
| 379 | fantom3_6430517C21 | fantom3_6430517C21 | -1.05958 | 4.03E-76 |
| 380 | NR_015492 | C230035I16Rik | -1.05902 | 1.02E-05 |
| 381 | fantom3_A430094C23 | fantom3_A430094C23 | -1.05902 | 1.02E-05 |
| 382 | NR_004434 | Rprl1 | -1.05744 | 4.17E-07 |
| 383 | fantom3_A530047A03 | fantom3_A530047A03 | -1.05656 | 0.000164 |
| 384 | fantom3_E030033J20 | fantom3_E030033J20 | -1.05636 | 1.93E-07 |
| 385 | fantom3_1110029I05 | fantom3_1110029I05 | -1.05511 | 1.66E-25 |
| 386 | NR_037773 | Tmem41a | -1.04922 | 8.33E-32 |
| 387 | fantom3_9430026M12 | fantom3_9430026M12 | -1.04914 | 8.26E-19 |
| 388 | fantom3_E130010O04 | fantom3_E130010O04 | -1.0463 | 0.000219 |
| 389 | fantom3_5730525L23 | fantom3_5730525L23 | -1.04551 | 4.63E-13 |
| 390 | fantom3_E330016E14 | fantom3_E330016E14 | -1.04517 | 2.17E-06 |
| 391 | fantom3_3010002K18 | fantom3_3010002K18 | -1.04364 | 1.38E-05 |
| 392 | fantom3_3830422K02 | fantom3_3830422K02 | -1.04359 | 1.65E-07 |
| 393 | fantom3_C430029M22 | fantom3_C430029M22 | -1.03863 | 1.07E-25 |
| 394 | fantom3_2310005L22 | fantom3_2310005L22 | -1.03832 | 1.65E-22 |
| 395 | fantom3_4732469H22 | fantom3_4732469H22 | -1.03633 | 4.06E-05 |
| 396 | fantom3_C430049B03 | fantom3_C430049B03 | -1.03207 | 1.76E-22 |
| 397 | fantom3_5830416C07 | fantom3_5830416C07 | -1.028 | 1.68E-27 |
| 398 | fantom3_A530088A10 | fantom3_A530088A10 | -1.02591 | 1.43E-30 |
| 399 | NR_027772 | Airn | -1.02569 | 7.73E-09 |
| 400 | fantom3_B230342L12 | fantom3_B230342L12 | -1.02485 | 7.62E-08 |
| 401 | fantom3_9630024D05 | fantom3_9630024D05 | -1.02047 | 1.36E-33 |
| 402 | fantom3_1700056E03 | fantom3_1700056E03 | -1.02029 | 1.01E-07 |
| 403 | fantom3_F830228J06 | fantom3_F830228J06 | -1.01982 | 4.70E-05 |
| 404 | fantom3_D530031N14 | fantom3_D530031N14 | -1.01878 | 0.000183 |
| 405 | NR_029468 | Snhg12 | -1.01755 | 1.38E-19 |
| 406 | fantom3_1700080F18 | fantom3_1700080F18 | -1.01636 | 4.79E-07 |
| 407 | fantom3_6720428G05 | fantom3_6720428G05 | -1.01481 | 3.76E-09 |
| 408 | fantom3_2810404D04 | fantom3_2810404D04 | -1.01289 | 6.30E-20 |
| 409 | fantom3_A230069A22 | fantom3_A230069A22 | -1.01226 | 0.000183 |
| 410 | fantom3_9530032E18 | fantom3_9530032E18 | -1.0118 | 8.49E-06 |
| 411 | fantom3_E330014N23 | fantom3_E330014N23 | -1.01096 | 2.16E-36 |
| 412 | fantom3_D030067C03 | fantom3_D030067C03 | -1.00839 | 2.90E-05 |
| 413 | fantom3_6330582G09 | fantom3_6330582G09 | -1.00721 | 3.42E-51 |
| 414 | fantom3_5330403E07 | fantom3_5330403E07 | -1.00348 | 4.59E-10 |
| 415 | fantom3_9130015G05 | fantom3_9130015G05 | -1.00244 | 8.61E-06 |
| 416 | fantom3_1500001C02 | fantom3_1500001C02 | -1.00214 | 2.13E-11 |
| 417 | fantom3_A130060J16 | fantom3_A130060J16 | -1.00078 | 9.75E-06 |
| 418 | fantom3_C630012A10 | fantom3_C630012A10 | 1.000978 | 1.44E-05 |
| 419 | fantom3_A430002D02 | fantom3_A430002D02 | 1.002776 | 0 |
| 420 | fantom3_8030403A17 | fantom3_8030403A17 | 1.003366 | 1.23E-06 |
| 421 | NR_015515 | 6430562O15Rik | 1.008766 | 0 |
| 422 | fantom3_6030478L11 | fantom3_6030478L11 | 1.008766 | 0 |
| 423 | fantom3_C130016P21 | fantom3_C130016P21 | 1.008804 | 4.49E-09 |
| 424 | fantom3_D930046M13 | fantom3_D930046M13 | 1.011521 | 0 |
| 425 | fantom3_4930558J22 | fantom3_4930558J22 | 1.017121 | 0 |
| 426 | fantom3_9530011I20 | fantom3_9530011I20 | 1.018649 | 1.33E-15 |
| 427 | fantom3_D830035A11 | fantom3_D830035A11 | 1.019145 | 0 |
| 428 | fantom3_9630012I08 | fantom3_9630012I08 | 1.020467 | 0 |
| 429 | fantom3_1010001E19 | fantom3_1010001E19 | 1.022981 | 0 |
| 430 | fantom3_D230004M03 | fantom3_D230004M03 | 1.023691 | 0.000202 |
| 431 | fantom3_E130215H24 | fantom3_E130215H24 | 1.026155 | 0 |
| 432 | fantom3_E030024E19 | fantom3_E030024E19 | 1.027238 | 3.86E-06 |
| 433 | fantom3_C230031I18 | fantom3_C230031I18 | 1.028263 | 9.65E-06 |
| 434 | fantom3_6430514H16 | fantom3_6430514H16 | 1.028379 | 0 |
| 435 | fantom3_4921528D17 | fantom3_4921528D17 | 1.031417 | 0 |
| 436 | fantom3_9930111E19 | fantom3_9930111E19 | 1.033641 | 4.66E-07 |
| 437 | fantom3_9630044E16 | fantom3_9630044E16 | 1.034935 | 2.22E-16 |
| 438 | fantom3_E030009P22 | fantom3_E030009P22 | 1.03995 | 2.51E-07 |
| 439 | fantom3_2610022A11 | fantom3_2610022A11 | 1.040179 | 0 |
| 440 | fantom3_A330077K16 | fantom3_A330077K16 | 1.047529 | 3.54E-06 |
| 441 | NR_045053 | 2610307P16Rik | 1.048122 | 6.60E-08 |
| 442 | fantom3_9630046K15 | fantom3_9630046K15 | 1.052427 | 1.36E-12 |
| 443 | fantom3_D830012D22 | fantom3_D830012D22 | 1.058728 | 0 |
| 444 | fantom3_6430400I16 | fantom3_6430400I16 | 1.059606 | 3.74E-13 |
| 445 | fantom3_1700010B09 | fantom3_1700010B09 | 1.05985 | 2.99E-08 |
| 446 | fantom3_D030072M03 | fantom3_D030072M03 | 1.062027 | 0 |
| 447 | fantom3_K530024H12 | fantom3_K530024H12 | 1.062148 | 0 |
| 448 | NR_027967 | Hhatl | 1.067054 | 0 |
| 449 | fantom3_C430005P17 | fantom3_C430005P17 | 1.072994 | 0 |
| 450 | fantom3_E030018F15 | fantom3_E030018F15 | 1.077524 | 0 |
| 451 | fantom3_6820448K18 | fantom3_6820448K18 | 1.078453 | 4.29E-07 |
| 452 | fantom3_8430406N05 | fantom3_8430406N05 | 1.083153 | 1.21E-07 |
| 453 | fantom3_E430001N13 | fantom3_E430001N13 | 1.083277 | 0 |
| 454 | fantom3_B130042E22 | fantom3_B130042E22 | 1.088357 | 6.63E-10 |
| 455 | fantom3_9630021P08 | fantom3_9630021P08 | 1.090428 | 2.78E-07 |
| 456 | fantom3_0710005G15 | fantom3_0710005G15 | 1.091897 | 5.55E-05 |
| 457 | fantom3_2900024I10 | fantom3_2900024I10 | 1.099215 | 2.66E-05 |
| 458 | fantom3_1110065P19 | fantom3_1110065P19 | 1.112357 | 9.94E-08 |
| 459 | fantom3_2610307P16 | fantom3_2610307P16 | 1.11257 | 9.29E-08 |
| 460 | fantom3_B930052M22 | fantom3_B930052M22 | 1.117357 | 2.18E-05 |
| 461 | fantom3_G730023M12 | fantom3_G730023M12 | 1.117547 | 0 |
| 462 | fantom3_4930568A15 | fantom3_4930568A15 | 1.121476 | 0 |
| 463 | fantom3_A230060L24 | fantom3_A230060L24 | 1.127116 | 3.96E-05 |
| 464 | NR_045054 | 2610307P16Rik | 1.127635 | 1.13E-08 |
| 465 | fantom3_4930568P13 | fantom3_4930568P13 | 1.132413 | 3.10E-06 |
| 466 | fantom3_D830027N09 | fantom3_D830027N09 | 1.134642 | 2.20E-07 |
| 467 | fantom3_A130076P06 | fantom3_A130076P06 | 1.137868 | 0.000189 |
| 468 | fantom3_7530404M11 | fantom3_7530404M11 | 1.138314 | 1.07E-12 |
| 469 | fantom3_A730069D24 | fantom3_A730069D24 | 1.140857 | 0.000199 |
| 470 | fantom3_4921524P08 | fantom3_4921524P08 | 1.143932 | 2.22E-16 |
| 471 | fantom3_9530001D12 | fantom3_9530001D12 | 1.144004 | 6.86E-13 |
| 472 | NR_045326 | A330009N23Rik | 1.144991 | 0.000133 |
| 473 | fantom3_D630028D06 | fantom3_D630028D06 | 1.146746 | 2.65E-05 |
| 474 | fantom3_F830221K10 | fantom3_F830221K10 | 1.147324 | 1.56E-05 |
| 475 | fantom3_9330151G08 | fantom3_9330151G08 | 1.14913 | 3.02E-05 |
| 476 | fantom3_7120429C13 | fantom3_7120429C13 | 1.154939 | 1.03E-08 |
| 477 | fantom3_G630062H16 | fantom3_G630062H16 | 1.165962 | 1.86E-10 |
| 478 | fantom3_9530036D08 | fantom3_9530036D08 | 1.176338 | 0 |
| 479 | fantom3_D230043I17 | fantom3_D230043I17 | 1.183051 | 0 |
| 480 | fantom3_4930578O05 | fantom3_4930578O05 | 1.184232 | 0 |
| 481 | fantom3_A730020G15 | fantom3_A730020G15 | 1.189468 | 0 |
| 482 | fantom3_5730405L21 | fantom3_5730405L21 | 1.191957 | 0 |
| 483 | fantom3_D030065H24 | fantom3_D030065H24 | 1.194053 | 7.29E-09 |
| 484 | fantom3_A730096M12 | fantom3_A730096M12 | 1.195763 | 4.86E-07 |
| 485 | fantom3_9630026O22 | fantom3_9630026O22 | 1.196314 | 0 |
| 486 | fantom3_4732454L13 | fantom3_4732454L13 | 1.197752 | 0 |
| 487 | fantom3_2700098C24 | fantom3_2700098C24 | 1.197928 | 0 |
| 488 | fantom3_A430083J21 | fantom3_A430083J21 | 1.199568 | 3.44E-06 |
| 489 | fantom3_B230343A10 | fantom3_B230343A10 | 1.199861 | 1.13E-12 |
| 490 | fantom3_5730435J12 | fantom3_5730435J12 | 1.209886 | 0.000199 |
| 491 | fantom3_A830073O21 | fantom3_A830073O21 | 1.211929 | 0 |
| 492 | fantom3_9830164E16 | fantom3_9830164E16 | 1.216066 | 1.80E-07 |
| 493 | fantom3_9630033M11 | fantom3_9630033M11 | 1.22217 | 6.29E-13 |
| 494 | fantom3_A530088N01 | fantom3_A530088N01 | 1.224746 | 0 |
| 495 | fantom3_3222402J19 | fantom3_3222402J19 | 1.227891 | 5.15E-13 |
| 496 | fantom3_2310024F14 | fantom3_2310024F14 | 1.229108 | 0 |
| 497 | fantom3_A630066O12 | fantom3_A630066O12 | 1.23523 | 0 |
| 498 | NR_029460 | 6530402F18Rik | 1.243984 | 1.48E-06 |
| 499 | fantom3_9530094C13 | fantom3_9530094C13 | 1.243988 | 1.48E-06 |
| 500 | fantom3_E130114J20 | fantom3_E130114J20 | 1.246668 | 9.71E-05 |
| 501 | fantom3_C130045B11 | fantom3_C130045B11 | 1.255923 | 0 |
| 502 | NR_039592 | Mir5130 | 1.258277 | 2.11E-10 |
| 503 | fantom3_C630024D06 | fantom3_C630024D06 | 1.2585 | 0 |
| 504 | fantom3_D630044L13 | fantom3_D630044L13 | 1.258566 | 0 |
| 505 | fantom3_9530076L14 | fantom3_9530076L14 | 1.259682 | 0 |
| 506 | fantom3_6430599K20 | fantom3_6430599K20 | 1.263172 | 0 |
| 507 | fantom3_C130061F03 | fantom3_C130061F03 | 1.264392 | 0 |
| 508 | fantom3_5330404B03 | fantom3_5330404B03 | 1.273222 | 0 |
| 509 | fantom3_E430020B19 | fantom3_E430020B19 | 1.276059 | 0.000145 |
| 510 | fantom3_1700119I11 | fantom3_1700119I11 | 1.28668 | 0 |
| 511 | NR_040659 | Gm5860 | 1.291872 | 1.28E-07 |
| 512 | fantom3_C030033F14 | fantom3_C030033F14 | 1.295586 | 2.18E-07 |
| 513 | fantom3_6430599O21 | fantom3_6430599O21 | 1.297159 | 1.02E-05 |
| 514 | fantom3_4932442G11 | fantom3_4932442G11 | 1.303698 | 5.12E-06 |
| 515 | fantom3_D930036H04 | fantom3_D930036H04 | 1.312066 | 8.02E-10 |
| 516 | fantom3_A330082J21 | fantom3_A330082J21 | 1.313674 | 4.45E-12 |
| 517 | NR_045556 | Cdh23 | 1.321094 | 8.72E-11 |
| 518 | fantom3_B230206C19 | fantom3_B230206C19 | 1.322259 | 0 |
| 519 | fantom3_A330042I21 | fantom3_A330042I21 | 1.323892 | 0.000102 |
| 520 | fantom3_C130012F17 | fantom3_C130012F17 | 1.3243 | 2.19E-05 |
| 521 | fantom3_3426401L16 | fantom3_3426401L16 | 1.32675 | 1.48E-06 |
| 522 | fantom3_B230399F14 | fantom3_B230399F14 | 1.334023 | 0 |
| 523 | fantom3_D830039A15 | fantom3_D830039A15 | 1.334466 | 5.73E-06 |
| 524 | fantom3_B230218A19 | fantom3_B230218A19 | 1.335245 | 2.98E-14 |
| 525 | fantom3_D930011C17 | fantom3_D930011C17 | 1.336839 | 2.65E-06 |
| 526 | NR_037984 | Gm19461 | 1.343463 | 2.22E-16 |
| 527 | fantom3_9530054H01 | fantom3_9530054H01 | 1.34699 | 4.76E-06 |
| 528 | fantom3_2010300P06 | fantom3_2010300P06 | 1.347255 | 1.90E-07 |
| 529 | fantom3_D030054N09 | fantom3_D030054N09 | 1.3492 | 0 |
| 530 | fantom3_9330142K14 | fantom3_9330142K14 | 1.355036 | 0.000182 |
| 531 | fantom3_G630042O16 | fantom3_G630042O16 | 1.355922 | 1.63E-08 |
| 532 | fantom3_I0C0049K08 | fantom3_I0C0049K08 | 1.36157 | 0 |
| 533 | fantom3_6030435F22 | fantom3_6030435F22 | 1.363785 | 5.71E-05 |
| 534 | fantom3_A230058M14 | fantom3_A230058M14 | 1.369513 | 2.73E-07 |
| 535 | fantom3_B930051C18 | fantom3_B930051C18 | 1.37825 | 3.34E-11 |
| 536 | fantom3_C130018M11 | fantom3_C130018M11 | 1.37953 | 0 |
| 537 | NR_037581 | Magix | 1.38291 | 0 |
| 538 | fantom3_E230021G05 | fantom3_E230021G05 | 1.38728 | 0 |
| 539 | fantom3_9930017F19 | fantom3_9930017F19 | 1.388359 | 1.05E-08 |
| 540 | fantom3_6430555I10 | fantom3_6430555I10 | 1.393546 | 1.48E-05 |
| 541 | fantom3_C030023J22 | fantom3_C030023J22 | 1.398534 | 0.000217 |
| 542 | fantom3_D830014B08 | fantom3_D830014B08 | 1.399008 | 5.00E-11 |
| 543 | fantom3_9530003H10 | fantom3_9530003H10 | 1.402676 | 0 |
| 544 | fantom3_4632428C04 | fantom3_4632428C04 | 1.405171 | 1.78E-05 |
| 545 | fantom3_A430062I15 | fantom3_A430062I15 | 1.406689 | 0 |
| 546 | fantom3_C130052N24 | fantom3_C130052N24 | 1.411411 | 0 |
| 547 | fantom3_F830014N22 | fantom3_F830014N22 | 1.412246 | 5.71E-05 |
| 548 | fantom3_9830146I01 | fantom3_9830146I01 | 1.424821 | 1.78E-15 |
| 549 | fantom3_C130066G01 | fantom3_C130066G01 | 1.429984 | 6.67E-05 |
| 550 | fantom3_9530007G09 | fantom3_9530007G09 | 1.43192 | 0 |
| 551 | fantom3_7530427C14 | fantom3_7530427C14 | 1.435569 | 1.04E-07 |
| 552 | fantom3_B230216K11 | fantom3_B230216K11 | 1.447691 | 0 |
| 553 | fantom3_D230046O15 | fantom3_D230046O15 | 1.459536 | 9.08E-09 |
| 554 | fantom3_E330005N08 | fantom3_E330005N08 | 1.459702 | 8.29E-05 |
| 555 | fantom3_2610016O05 | fantom3_2610016O05 | 1.463373 | 8.00E-05 |
| 556 | fantom3_C230083H17 | fantom3_C230083H17 | 1.466281 | 0.000221 |
| 557 | fantom3_D130026H02 | fantom3_D130026H02 | 1.466391 | 0.000173 |
| 558 | fantom3_D830011I19 | fantom3_D830011I19 | 1.468347 | 2.23E-08 |
| 559 | fantom3_D130097H03 | fantom3_D130097H03 | 1.469846 | 0.00012 |
| 560 | fantom3_A230004P08 | fantom3_A230004P08 | 1.473223 | 4.91E-08 |
| 561 | fantom3_9330161B17 | fantom3_9330161B17 | 1.474654 | 2.48E-10 |
| 562 | fantom3_D130008B01 | fantom3_D130008B01 | 1.478695 | 7.59E-08 |
| 563 | fantom3_C130040E24 | fantom3_C130040E24 | 1.482294 | 0.000114 |
| 564 | fantom3_C130035N23 | fantom3_C130035N23 | 1.487543 | 3.93E-06 |
| 565 | fantom3_A130037H21 | fantom3_A130037H21 | 1.493009 | 2.88E-07 |
| 566 | fantom3_C130042M10 | fantom3_C130042M10 | 1.49349 | 2.22E-16 |
| 567 | fantom3_2310041C05 | fantom3_2310041C05 | 1.497146 | 5.66E-09 |
| 568 | fantom3_D030034A15 | fantom3_D030034A15 | 1.514286 | 1.66E-06 |
| 569 | fantom3_D930033I06 | fantom3_D930033I06 | 1.526467 | 4.16E-09 |
| 570 | fantom3_A530056D08 | fantom3_A530056D08 | 1.548719 | 0.000114 |
| 571 | fantom3_D630034K06 | fantom3_D630034K06 | 1.553949 | 4.43E-05 |
| 572 | fantom3_A230109G23 | fantom3_A230109G23 | 1.55783 | 0 |
| 573 | fantom3_9930004G02 | fantom3_9930004G02 | 1.560607 | 0.000114 |
| 574 | fantom3_C730040C05 | fantom3_C730040C05 | 1.564531 | 8.07E-09 |
| 575 | fantom3_B430215G09 | fantom3_B430215G09 | 1.571509 | 6.31E-09 |
| 576 | fantom3_1700020G04 | fantom3_1700020G04 | 1.581799 | 0 |
| 577 | fantom3_A230066L14 | fantom3_A230066L14 | 1.596172 | 3.29E-06 |
| 578 | fantom3_2310008C07 | fantom3_2310008C07 | 1.596591 | 2.44E-11 |
| 579 | fantom3_D830038L22 | fantom3_D830038L22 | 1.599222 | 0 |
| 580 | fantom3_A330032P22 | fantom3_A330032P22 | 1.600308 | 4.05E-07 |
| 581 | fantom3_2310003D02 | fantom3_2310003D02 | 1.603286 | 4.36E-11 |
| 582 | NR_033631 | 4632428C04Rik | 1.608454 | 0 |
| 583 | fantom3_D830002B11 | fantom3_D830002B11 | 1.608454 | 0 |
| 584 | fantom3_C230052C06 | fantom3_C230052C06 | 1.608461 | 5.08E-07 |
| 585 | fantom3_B430312A05 | fantom3_B430312A05 | 1.615983 | 0 |
| 586 | fantom3_6230431N17 | fantom3_6230431N17 | 1.616726 | 2.11E-14 |
| 587 | fantom3_D830044O10 | fantom3_D830044O10 | 1.634179 | 4.90E-05 |
| 588 | fantom3_C730029A08 | fantom3_C730029A08 | 1.651556 | 0 |
| 589 | fantom3_A230073G04 | fantom3_A230073G04 | 1.660935 | 0 |
| 590 | fantom3_F830038G05 | fantom3_F830038G05 | 1.677554 | 0.000145 |
| 591 | fantom3_5830408B19 | fantom3_5830408B19 | 1.691129 | 2.11E-14 |
| 592 | fantom3_6530404P05 | fantom3_6530404P05 | 1.69243 | 0.000226 |
| 593 | fantom3_2010208B10 | fantom3_2010208B10 | 1.698931 | 2.41E-05 |
| 594 | fantom3_A530048B07 | fantom3_A530048B07 | 1.709924 | 0.000192 |
| 595 | fantom3_A330001P17 | fantom3_A330001P17 | 1.711319 | 3.19E-05 |
| 596 | fantom3_A530063B08 | fantom3_A530063B08 | 1.712057 | 3.98E-09 |
| 597 | fantom3_D430007O09 | fantom3_D430007O09 | 1.720711 | 0 |
| 598 | fantom3_A130068E22 | fantom3_A130068E22 | 1.720935 | 1.78E-05 |
| 599 | fantom3_C530032N24 | fantom3_C530032N24 | 1.726258 | 5.20E-11 |
| 600 | fantom3_1110058D09 | fantom3_1110058D09 | 1.7496 | 0 |
| 601 | fantom3_6330444A09 | fantom3_6330444A09 | 1.751599 | 0 |
| 602 | fantom3_F730311O21 | fantom3_F730311O21 | 1.756387 | 6.28E-06 |
| 603 | fantom3_D430036F12 | fantom3_D430036F12 | 1.757367 | 9.84E-06 |
| 604 | fantom3_9630007F23 | fantom3_9630007F23 | 1.771601 | 2.88E-09 |
| 605 | fantom3_8030487G03 | fantom3_8030487G03 | 1.777951 | 0.00014 |
| 606 | fantom3_A530082M10 | fantom3_A530082M10 | 1.78458 | 1.54E-05 |
| 607 | fantom3_1110066J06 | fantom3_1110066J06 | 1.79395 | 2.28E-10 |
| 608 | fantom3_5430431D22 | fantom3_5430431D22 | 1.795959 | 8.12E-09 |
| 609 | fantom3_D830011L12 | fantom3_D830011L12 | 1.802182 | 7.37E-08 |
| 610 | fantom3_A530095I10 | fantom3_A530095I10 | 1.802599 | 0 |
| 611 | fantom3_A330090B06 | fantom3_A330090B06 | 1.806212 | 9.12E-06 |
| 612 | fantom3_9830141C07 | fantom3_9830141C07 | 1.817644 | 8.69E-05 |
| 613 | fantom3_D030004O22 | fantom3_D030004O22 | 1.819639 | 7.13E-09 |
| 614 | fantom3_D130059B14 | fantom3_D130059B14 | 1.831879 | 7.37E-08 |
| 615 | fantom3_5033414K04 | fantom3_5033414K04 | 1.844356 | 8.69E-05 |
| 616 | NR_027627 | Kcnk2 | 1.844934 | 2.89E-15 |
| 617 | fantom3_D930015L03 | fantom3_D930015L03 | 1.854357 | 1.13E-12 |
| 618 | fantom3_3732412A05 | fantom3_3732412A05 | 1.859514 | 6.44E-10 |
| 619 | fantom3_A430070E15 | fantom3_A430070E15 | 1.866197 | 4.49E-06 |
| 620 | fantom3_9430096B02 | fantom3_9430096B02 | 1.866244 | 4.79E-05 |
| 621 | fantom3_I830028F20 | fantom3_I830028F20 | 1.882796 | 3.40E-07 |
| 622 | NR_033261 | Gm14492 | 1.923222 | 0 |
| 623 | fantom3_9530045K08 | fantom3_9530045K08 | 1.933316 | 6.26E-06 |
| 624 | fantom3_6430543G08 | fantom3_6430543G08 | 1.94284 | 0.00014 |
| 625 | fantom3_4732429D16 | fantom3_4732429D16 | 1.965468 | 5.66E-06 |
| 626 | fantom3_B230372B02 | fantom3_B230372B02 | 1.996835 | 0.000175 |
| 627 | fantom3_1110002O04 | fantom3_1110002O04 | 1.999814 | 0.000114 |
| 628 | fantom3_9630045C05 | fantom3_9630045C05 | 2.000497 | 0.00019 |
| 629 | fantom3_D830006B11 | fantom3_D830006B11 | 2.001288 | 4.71E-13 |
| 630 | fantom3_C130031M05 | fantom3_C130031M05 | 2.011032 | 3.28E-05 |
| 631 | fantom3_5830407M18 | fantom3_5830407M18 | 2.015347 | 0 |
| 632 | fantom3_9330010F12 | fantom3_9330010F12 | 2.037519 | 0.00019 |
| 633 | fantom3_3526401B18 | fantom3_3526401B18 | 2.038545 | 1.20E-06 |
| 634 | fantom3_4932443D20 | fantom3_4932443D20 | 2.047981 | 5.90E-05 |
| 635 | fantom3_5830402E17 | fantom3_5830402E17 | 2.056676 | 9.68E-05 |
| 636 | fantom3_6330410N15 | fantom3_6330410N15 | 2.069048 | 0.000114 |
| 637 | NR_040260 | 2210408F21Rik | 2.107958 | 0.000114 |
| 638 | NR_015539 | 9630013A20Rik | 2.120159 | 0.00019 |
| 639 | fantom3_9630023K13 | fantom3_9630023K13 | 2.120159 | 0.00019 |
| 640 | fantom3_9630013A20 | fantom3_9630013A20 | 2.157256 | 2.36E-05 |
| 641 | NR_040259 | 2210408F21Rik | 2.208135 | 2.36E-05 |
| 642 | fantom3_9330167E06 | fantom3_9330167E06 | 2.223794 | 0.000202 |
| 643 | fantom3_A730084J12 | fantom3_A730084J12 | 2.247796 | 4.15E-06 |
| 644 | fantom3_F630014I20 | fantom3_F630014I20 | 2.261244 | 2.29E-05 |
| 645 | NR_040262 | 2210408F21Rik | 2.268421 | 2.36E-05 |
| 646 | fantom3_B230032O15 | fantom3_B230032O15 | 2.275782 | 0 |
| 647 | fantom3_G630029K15 | fantom3_G630029K15 | 2.288021 | 0 |
| 648 | NR_040258 | 2210408F21Rik | 2.308395 | 4.69E-06 |
| 649 | fantom3_A430027H14 | fantom3_A430027H14 | 2.329156 | 6.72E-08 |
| 650 | fantom3_4732401I11 | fantom3_4732401I11 | 2.33002 | 0 |
| 651 | fantom3_5031405M08 | fantom3_5031405M08 | 2.35852 | 0 |
| 652 | fantom3_9930010F05 | fantom3_9930010F05 | 2.36489 | 0 |
| 653 | NR_040261 | 2210408F21Rik | 2.370852 | 2.36E-05 |
| 654 | NR_040257 | 2210408F21Rik | 2.372232 | 2.77E-06 |
| 655 | fantom3_D630048K14 | fantom3_D630048K14 | 2.386542 | 0 |
| 656 | fantom3_A930039P05 | fantom3_A930039P05 | 2.394298 | 0 |
| 657 | fantom3_D930007A20 | fantom3_D930007A20 | 2.43696 | 4.01E-05 |
| 658 | fantom3_D930005L05 | fantom3_D930005L05 | 2.449702 | 1.62E-06 |
| 659 | fantom3_2310007J06 | fantom3_2310007J06 | 2.461489 | 2.12E-08 |
| 660 | fantom3_D830028G10 | fantom3_D830028G10 | 2.526868 | 0 |
| 661 | fantom3_9130004I19 | fantom3_9130004I19 | 2.534866 | 2.36E-05 |
| 662 | fantom3_B230365N22 | fantom3_B230365N22 | 2.543659 | 3.17E-10 |
| 663 | fantom3_D130050H17 | fantom3_D130050H17 | 2.596146 | 0.000103 |
| 664 | fantom3_6030407P20 | fantom3_6030407P20 | 2.604686 | 6.91E-05 |
| 665 | fantom3_A930025H08 | fantom3_A930025H08 | 2.626115 | 3.70E-12 |
| 666 | fantom3_A730046I10 | fantom3_A730046I10 | 2.626178 | 7.21E-12 |
| 667 | fantom3_A330052J17 | fantom3_A330052J17 | 2.653846 | 3.12E-11 |
| 668 | fantom3_6030430G11 | fantom3_6030430G11 | 2.689564 | 1.42E-13 |
| 669 | fantom3_D430020M16 | fantom3_D430020M16 | 2.699525 | 0 |
| 670 | fantom3_9230104K02 | fantom3_9230104K02 | 2.738828 | 0.000103 |
| 671 | fantom3_9330183L06 | fantom3_9330183L06 | 2.815748 | 0 |
| 672 | NR_102366 | AW112010 | 2.833716 | 4.64E-07 |
| 673 | fantom3_A530032D21 | fantom3_A530032D21 | 2.874953 | 6.63E-08 |
| 674 | fantom3_A930018M24 | fantom3_A930018M24 | 2.928164 | 0 |
| 675 | fantom3_1300003O18 | fantom3_1300003O18 | 2.972988 | 5.71E-07 |
| 676 | fantom3_A730091J11 | fantom3_A730091J11 | 3.045763 | 0.000152 |
| 677 | fantom3_A230064C13 | fantom3_A230064C13 | 3.068417 | 6.62E-13 |
| 678 | fantom3_D830007H18 | fantom3_D830007H18 | 3.069193 | 1.20E-07 |
| 679 | fantom3_D830032O22 | fantom3_D830032O22 | 3.333838 | 8.13E-05 |
| 680 | fantom3_4732418A04 | fantom3_4732418A04 | 3.345622 | 2.67E-08 |
| 681 | fantom3_9430057E02 | fantom3_9430057E02 | 3.493831 | 2.31E-05 |
| 682 | fantom3_9630013F18 | fantom3_9630013F18 | 3.587597 | 1.92E-10 |
| 683 | fantom3_2210409C20 | fantom3_2210409C20 | 4.973428 | 0 |
| 684 | fantom3_2310015A16 | fantom3_2310015A16 | Inf | 2.95E-07 |
| 685 | fantom3_B230352I09 | fantom3_B230352I09 | #NAME? | 2.20E-05 |
